# Brønsted/Lewis acid sites synergistically promote the initial C–C bond formation in the MTO reaction†
†Electronic supplementary information (ESI) available: Detailed zeolite structures, the reaction mechanisms for the traditional methane–formaldehyde pathway and the ethene formation routes, the energy profiles for C–C bond formation over ZSM-5 and SSZ-13, the optimized structures for all the intermediates and transition states, the geometric parameters for all the intermediates and transition states. See DOI: 10.1039/c8sc02302f


**DOI:** 10.1039/c8sc02302f

**Published:** 2018-06-27

**Authors:** Yueying Chu, Xianfeng Yi, Chengbin Li, Xianyong Sun, Anmin Zheng

**Affiliations:** a State Key Laboratory of Magnetic Resonance and Atomic and Molecular Physics , National Center for Magnetic Resonance in Wuhan , Key Laboratory of Magnetic Resonance in Biological Systems , Wuhan Institute of Physics and Mathematics , Chinese Academy of Sciences , Wuhan 430071 , P. R. China . Email: zhenganm@wipm.ac.cn

## Abstract

The methanol-to-olefin (MTO) reaction is an active field of research due to conflicting mechanistic proposals for the initial carbon–carbon (C–C) bond formation. Herein, a new methane–formaldehyde pathway, a Lewis acid site combined with a Brønsted acid site in zeolite catalysts can readily activate dimethyl ether (DME) to form ethene, is identified theoretically. The mechanism involves a hydride transfer from Al–OCH_3_ on the Lewis acid site to the methyl group of the protonated methanol molecule on the adjacent Brønsted acid site leading to synchronous formation of methane and Al–COH_2_^+^ (which can be considered as formaldehyde (HCHO) adsorbed on the Al^3+^ Lewis acid sites). The strong electrophilic character of the Al–COH_2_^+^ intermediate can strongly accelerate the C–C bond formation with CH_4_, as indicated by the significant decrease of activation barriers in the rate-determining-step of the catalytic processes. These results highlight a synergy of extra-framework aluminum (EFAl) Lewis and Brønsted sites in zeolite catalysts that facilitates initial C–C bond formation in the initiation step of the MTO reaction *via* the Al–COH_2_^+^ intermediate.

## Abstract 2

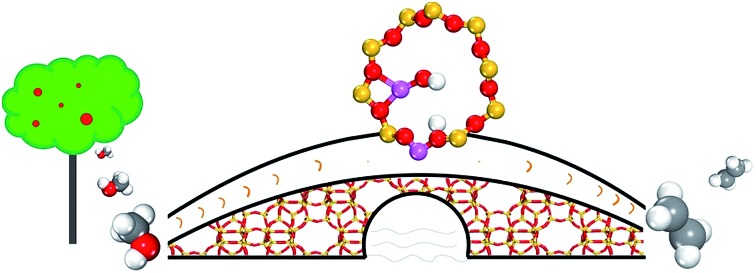
The Lewis acid site combined with a Brønsted acid site in zeolite catalysts facilitates first C–C bond formation in the initiation step of the MTO reaction.

An industrial breakthrough stemmed from the discovery of the methanol to olefin (MTO) process, which allowed the catalytic conversion of methanol to ethylene and propylene by zeolites.^1^ This process constitutes an alternative route to light alkenes not relying on crude oil. The mechanism of this process has become a matter of intense debate and investigation both in industry and academia. On the basis of experimental and theoretical studies, two types of mechanisms, the direct one, and the hydrocarbon pool (HCP) one, have been proposed to explain C–C bond formation. The popularly accepted one is the HCP mechanism, in which carbenium species have been confirmed as the active species,^2^–^4^ and a complete catalytic cycle combining theory and experiment has been put forward for HZSM-5 and HSAPO-34 zeolites.^5^,^6^ Aromatics like polymethylbenzenes (MBs) or olefins like higher olefins represent two kinds of important HCP species during the process of MTO conversion.^7^–^12^ However, the active sites and the formation mechanism of the initial C–C bond in the induction period, has remained a controversial issue. More than 20 mechanisms have been put forward to explain the formation of the initial C–C bond with participation of various reactive intermediates such as oxonium ylides, carbocations, carbenes and free radicals catalyzed by Brønsted acid sites of zeolite catalysts.^13^–^15^ It has been evaluated by theoretical calculations that all the proposed direct mechanisms, *e.g.* carbene, oxonium ylide and methane–formaldehyde mechanisms were inhibitive of the C–C bond formation on account of their high activation barriers (>44 kcal mol^–1^).^16^ Recently, several new mechanisms that are responsible for the initial C–C bond formation were proposed. Lercher and Weckhuysen *et al.* proposed that methyl acetate was the intermediate responsible for the initial C–C formation during the MTO reaction.^17^,^18^ Fan and coworkers proposed a route involving methoxymethyl cation (CH_3_OCH_2_^+^) intermediates, in which the barrier for initial C–C formation has a low activation energy (<39.0 kcal mol^–1^) over the Brønsted acid sites of HSAPO-34 and HZSM-5 zeolites.^19^,^20^ Besides the Brønsted acid catalysis, Copéret and Sautet *et al.* demonstrated that the surface Lewis acid sites on γ-Al_2_O_3_ also readily activate dimethyl ether (DME) to yield alkenes involving an Al-oxonium ion intermediate with a relatively low barrier (38.0 kcal mol^–1^).^21^ Recently, Liu *et al.* obtained some new insights into the initial C–C bond formation by using *in situ* solid-state NMR.^22^ They suggested that a surface methyleneoxy-analogue was the crucial intermediate for the initial C–C bond formation and the C–C bond direct formation *via* an interesting synergetic mechanism, involving C–H bond breakage and C–C bond coupling during the initial methanol reaction in the chemical environment of zeolite catalysis.

It's well known that a mild hydrothermal/thermal treatment usually results in a partial release of aluminum from a zeolite framework and causes formation of extra-framework aluminum (EFAl) species. Our previous NMR studies revealed that the formed EFAl species and the Brønsted site could be adjacent^23^–^25^ Additionally, White *et al.* also demonstrated that the Brønsted site and the extra-lattice Al–OH species are adjacent by using the 2D exchange NMR experiment, and a synergistic effect of Lewis sites near Brønsted bridging acid sites (BAS) existed.^26^ Inspired by the pioneering work by Hutchings and Hirao that HCHO and CH_4_ could be formed during methanol transformation over the Brønsted site of ZSM-5 zeolite and the reaction pathway proposed by Copéret that the surface Lewis acid sites on γ-Al_2_O_3_ readily activate dimethyl ether (DME) to yield alkenes, a new methane–formaldehyde mechanism associated with Brønsted acid and Lewis acid (*i.e.*, EFAl species) sites in the zeolite catalysts for the initial C–C bond formation is proposed in this contribution.^15^,^21^,^27^ As shown in Scheme 1, due to the synergy of the Brønsted acid/Lewis acid sites (BAS/LAS), the newly proposed mechanism differs from the conventional methane–formaldehyde mechanism route at the isolated Brønsted acid site and the reaction over γ-Al_2_O_3_. For example, the initial DME activation occurs at the Brønsted acid site and the Al–OCH_2_^+^ is responsible for the initial C–C bond formation. A large number of experimental results have demonstrated that CH_4_ was the first product during the methanol transformation.^15^,^28^ It is therefore clear that any mechanism proposed for the formation of the initial C–C bond must also account for the formation of methane in agreement with the experimental observations. The new route proposed here reveals the formation of the CH_4_ intermediate, in agreement with the previous work.^15^ Various mononuclear oxo aluminum cations (*i.e.*, AlO^+^, Al(OH)_2_^+^, and AlOH^2+^) and neutral species (*i.e.*, AlOOH and Al(OH)_3_) are the possible EFAl species in the zeolites as confirmed by NMR experiments.^23^,^24^ Besides the mononuclear EFAl, the previous DFT calculation study by Pidko showed that the multinuclear EFAl species also could be formed in Y zeolite.^29^ Compared with Y zeolite, ZSM-5 possesses a high Si/Al ratio, which significantly prevents the mononuclear EFAl condensation to multinuclear EFAl species during the hydrothermal/thermal treatment. Therefore, only five possible mononuclear EFAl species adjacent to the Brønsted site are systematically investigated to explore the possible active sites and detailed reaction mechanisms for the C–C bond formation over the ZSM-5 zeolite catalyst.

**Scheme 1:**
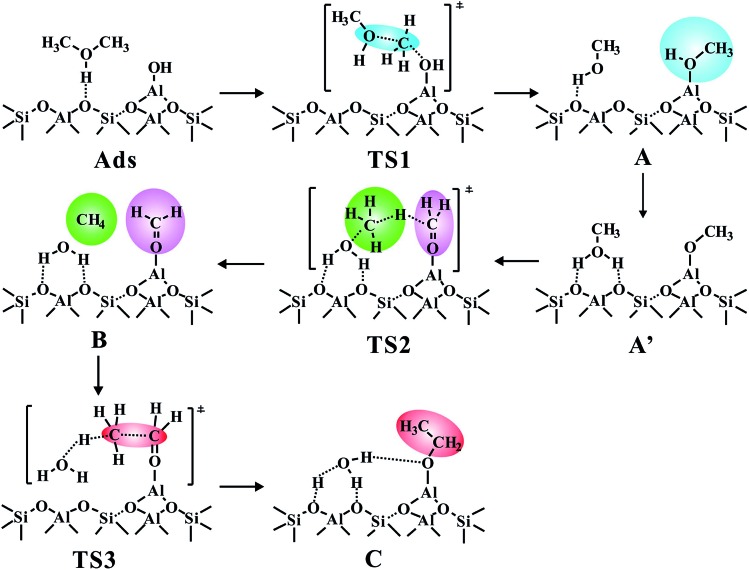
The newly proposed mechanism for the C–C bond formation at the synergistical BAS/LAS sites over zeolite catalysts. (Ads, represents the adsorbed DME; A, represents Al–OH-bound methyl (Al–OHCH_3_); A′, represents the Al-bound methoxide (Al–OCH_3_); B, represents the Al–OCH_2_^+^ intermediate; C, represents the Al-bound ethoxide (Al–O–CH_2_CH_3_); TS, represents the transition state).

## Calculation method

ZSM-5 and SSZ-13 zeolites are represented by 72T and 74T models, respectively, which were extracted from their crystallographic structural data.^30^ The 72T contains the complete double 10-MR intersection pores of ZSM-5 zeolite. The 74T SSZ-13 model includes two complete cages connected *via* an 8-MR window. The terminal Si–H was fixed at a bond length of 1.47 Å, oriented along the direction of the corresponding Si–O bond. Based on the previous studies, the Si_12_–O_24_(H)–Al_12_ and Si_1_–O_2_(H)–Al_1_ were chosen as the acid site positions for H-ZSM-5 and H-SSZ-13, respectively. It's theoretically demonstrated that the terminal oxygen (Al=O) atoms were not favored, but were prone to protonation to form EFAl hydroxy groups. For AlO^+^/HZSM-5, a spontaneous intramolecular acidic proton transfer readily occurred and resulted in the proton bound to the oxygen atom of the Al=O and resulted in AlOH^2+^ structure formation in HZSM-5 zeolite in the structure optimization, and a similar tendency was also observed in USY zeolite.^31^ For AlO^+^/HSSZ-13, AlOOH/HSSZ-13 and AlOOH/HZSM-5 with terminal oxygen (Al=O) atoms, the intramolecular proton transfer produced more stable structures as well. As indicated in Fig. S1,† the Gibbs free energies of the AlOH^2+^ at SSZ-13 (–29.4 kcal mol^–1^), Al(OH)_2_^+^ at SSZ-13 (–62.3 kcal mol^–1^) and Al(OH)_2_^+^ at ZSM-5 (–50.9 kcal mol^–1^) were much lower than those of the corresponding forms with separated terminal Al=O and BAS sites at 573 K. It's noteworthy that these AlOH^2+^ and Al(HO)_2_^+^ were the isolated AlOH^2+^ and Al(OH)_2_^+^ EFAl species since the nearby BAS have been consumed. In contrast to EFAl that contained a terminal Al=O group, the other EFAls (*i.e.*, AlOH^2+^, Al(OH)_2_^+^, Al(OH)_3_) were stable with the adjacent BAS in the ZSM-5 and SSZ-13 zeolites, and can be considered as AlOH^2+^/BAS, Al(OH)_2_^+^/BAS, and Al(OH)_3_/BAS models. Thus, the five EFAL/BAS structures over the two zeolites were modeled as AlOH^2+^/BAS (Fig. S2a and S2f†), Al(OH)_2_^+^/BAS (Fig. S2c and S2h†), Al(OH)_3_/BAS (Fig. S2d and S2i†) and isolated AlOH^2+^ (Fig. S2b and S2g†) and Al(OH)_2_^+^ (Fig. S2e and S2j†) inside the two zeolite frameworks.

In order to maintain the electrical neutrality of calculated models, 1 or 2 framework Al atoms were used. This method has been extensively used in theoretical calculations to investigate the dealumination process and the effect of EFAl species on the acidity of zeolites.^31^–^33^ The previous studies have demonstrated that Al–O–(Si–O)_2_–Al exists in Si-rich H-ZSM-5 zeolites.^34^ Additionally, the multiple Brønsted acid protons (multiple framework Al atoms) in HZSM-5 zeolite have been directly observed by White's group using a combination of 1D and 2D MAS NMR experiments.^26^ Thus, two framework Al atoms separated by two framework Si sites used in this work for HZSM-5 zeolite are in agreement with the experimental structures.

In this work, the active site atoms and the adsorbed hydrocarbon complex were treated as the high-level layer (see Fig. S1†), while the rest of the frameworks were treated as the low-level layer. To retain the structural integrities of the modeled zeolite, partial structure optimizations of the 72T and 74T clusters were performed by relaxing the atoms in the high-level layer while keeping the rest of atoms fixed at their crystallographic positions. All the TS structures are found by the QST3 method using the Gaussian program. Then based on the imaginary vibrational model of the optimized TS, we adjusted the positions of the vibrational atoms slightly along the calculated reaction coordinate in the two directions toward the reactant and the product, respectively, and finally optimized the resulting structures to the minimum structures. These methods have been widely employed in other previous theoretical studies.

A combined theoretical approach, namely ONIOM (ωb97xd/6-31G(d,p): am1) was used for the geometry optimization of adsorption states and transition states (TS). The ωB97XD hybrid density function, combined with 6-31G(d,p) basis sets, was employed for the energy calculation. This method was a recently developed long-range-corrected hybrid functional by Chai and Head-Gordon, which implicitly accounted for empirical dispersion and could describe long-range dispersion interactions well with respect to the traditional density functional theory methods.^35^ This functional was also recently found to perform very well for the description of adsorption and reactions on zeolites. Since the AM1 method is believed to underestimate the low level interaction energies, all energies reported herein were predicted at the ωB97XD/6-31G(d, p) level based on the optimized structures. The combined method could reproduce the experimental results obtained on MTO zeolite catalysts.^36^

The harmonic frequency calculations employing a partial Hessian vibrational analysis (PHVA),^37^ including the high layer active acid sites and organic species were performed to check whether the stationary points found exhibit the appropriate number of imaginary frequencies. In frequency calculations, besides the atoms in the high-level layer and the organic fragment, the constraints of the zeolite framework were also kept the same as in geometry optimizations, so that only one imaginary frequency would be observed for transition state points and none for minima. The Gibbs free energies at 573 K were then calculated from harmonic frequencies.

## Results and disscusion

### The initial C–C bond formation over ZSM-5 zeolite

**Fig. 1:**
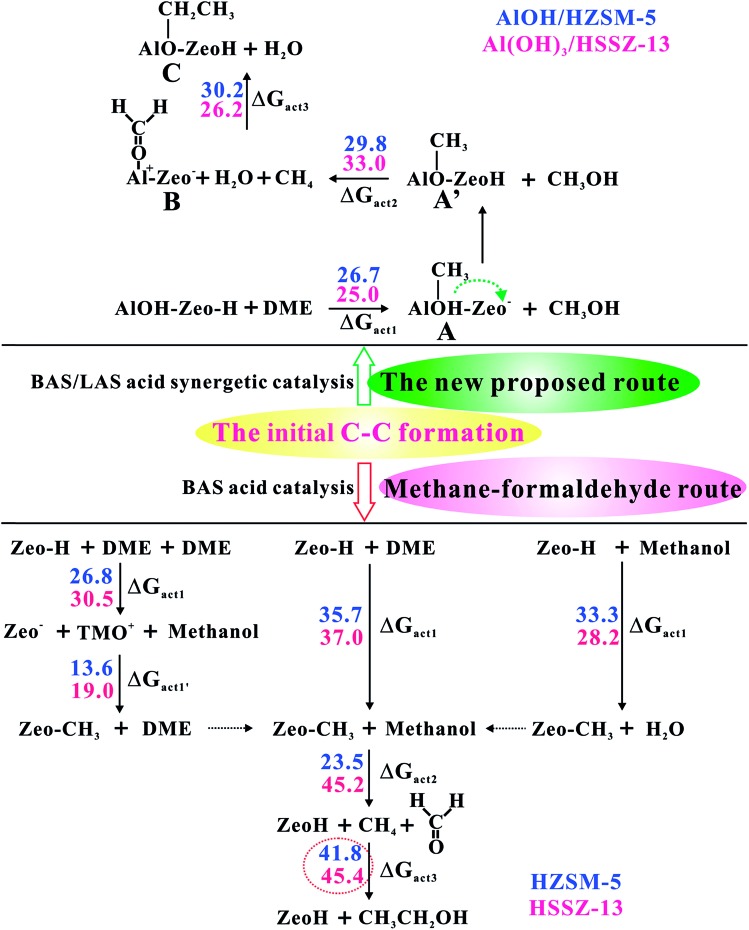
The conventional and newly proposed methane–formaldehyde routes at the Brønsted acid site and synergistical BAS/LAS sites for the C–C bond formation in the MTO reaction over the AlOH/HZSM-5 site and Al(OH)_3_/HSSZ-13 catalysts. The Gibbs free energy barriers (Δ*G*_act_, in kcal mol^–1^) for each step have been listed at 573 K. The detailed reaction routes are shown in Scheme 1 and S1† (A, represents the Al–OH-bound methyl (Al–OHCH_3_); A′, represents the Al-bound methoxide (Al–OCH_3_); B, represents Al–OCH_2_^+^ intermediate; C, represents Al-bound ethoxide (Al–O–CH_2_CH_3_)).

Compared with methanol, dimethyl ether (DME) is more suitable for exploring the direct C–C bond formation route due to its higher reactivity in the initial MTO process. The calculated results have demonstrated the feasible formation of DME during the MTO reaction with the energy barriers of 15.4 kcal mol^–1^ for ZSM-5 and 28.0 kcal mol^–1^ for SSZ-13 zeolite (the optimized TS structure, see Fig. S3†) which is in agreement with the experimental results that DME could easily be produced at the initial stage of the MTO reaction.^19^ The DME adsorption on the five active sites is an entropy reduction step, and the entropy losses are *ca.* –34.0 to –46.0 cal K^–1^ mol^–1^ for all five sites accompanied by the adsorption Gibbs free energies (Δ*G*_ads_) in the range of 2.3 to –23.2 kcal mol^–1^ (see Table S1†). It's apparently observed that DME is more readily adsorbed (Δ*G*_ads_ = –23.2 kcal mol^–1^) at AlOH/HZSM-5 among the five active centers and the optimized adsorption structure is provided in Fig. S4.† Thus, the C–C bond formation from the most stable state of DME adsorbed on the AlOH/BAS center, will be discussed in detail (Fig. 1 and S5†). In this case, the AlOH eases the CH_3_ migration of the protonated DME to produce AlOCH_3_ and leave a methanol molecule adsorbed on the conjugated alkaline oxygen center around the Brønsted acid site, *via* a barrier of 26.7 kcal mol^–1^. The lower energy barrier demonstrates that the Al–OCH_3_ intermediate could be readily generated, in agreement with Sautet's work that Al–OCH_3_ could be formed at the AlOH site during the DME transformation.^21^ Then, the generated methanol molecule allows abstraction of a hydride from the CH_3_ group in AlOCH_3_, generating methane and an Al–OCH_2_^+^ intermediate (B).

As illustrated in Fig. 1, the intermediate B will be formed with a barrier of 29.8 kcal mol^–1^. It's interesting to note that Al–COH_2_^+^ (B) can be considered as formaldehyde (HCHO) adsorbed on the Al^3+^ Lewis acid sites. Thus, the newly proposed mechanism is an analogous methane–formaldehyde route. It's revealed experimentally by Morton *et al.* that the C–H bond of alkoxide species could be weakened and lead to aldehyde group formation based on the lower CH stretching frequencies relative to the neutral alkanol molecule by using IR multiple photon dissociation (IRMPD) spectra.^38^ In this work, the low energy barrier for the Al–COH_2_^+^ formation from Al–OCH_3_ (Al methoxide) through TS2 is in good agreement with this experimental result. Additionally, Lercher *et al.* also demonstrated that the LAS sites could promote the yield of HCHO in ZSM-5 zeolite by hydride transfer.^39^ As a comparison, the methane–formaldehyde route for the C–C bond formation on the BAS is also investigated (see Fig. 1). In this mechanism, the surface CH_3_ attached to the hydrogen in the methyl group of methanol to form methane and HCHO, and subsequently, the methane reacts with HCHO to form ethanol (see Scheme S1†). As shown in Fig. 1, the C–C bond formation on the zeolite BAS site is strongly prohibited in the methane–formaldehyde mechanism due to the relatively high barrier (>40 kcal mol^–1^), which has also been illustrated in the previous work.^16^ However, due to the synergy of the BAS/LAS, the newly proposed mechanism of the C–C bond formation differs from the conventional one. Under synergistical BAS/LAS conditions, the strong electrophilic character of the Lewis acid site facilitates the addition reaction between the Al–OCH_2_^+^ and CH_4_ molecules, which leads to the C–C bond formation with the barrier decreasing to 30.2 kcal mol^–1^.

**Fig. 2:**
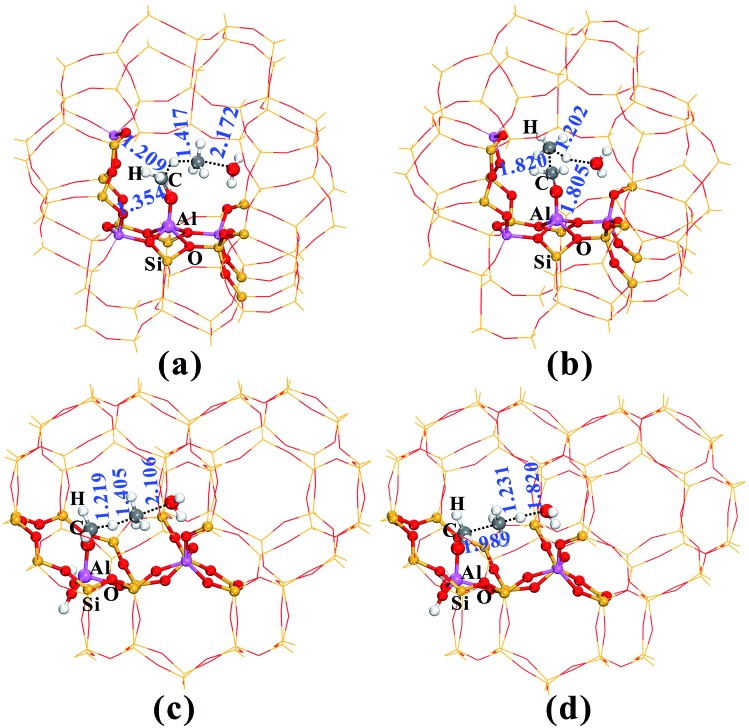
The optimized structures of the TS for AlOCH_2_^+^ (a and c) and C–C (b and d) bond formation over the synergistical AlOH/HZSM-5 (a and b) and Al(OH)_3_/HSSZ-13 (c and b) sites in the zeolite catalysts. The main geometric parameters are given in Å.

Fig. 2 provides the transition state structures of the concerted reactions for Al–COH_2_^+^ (Fig. 2a) and C–C bond (Fig. 2b) formation over the AlOH/BAS site in ZSM-5, and the complete structural change during the C–C bond formation is shown in Fig. S6 and Table S2†. For the Al–OCH_2_^+^ formation, the generated methanol molecule allows abstraction of a hydride from AlOCH_3_ to generate methane, Al–OCH_2_^+^ oxonium and H_2_O. The oxonium species is characterized by a C–O distance equal to 1.354 Å at the transition-state, while the distance of the newly formed C–H bond is equal to 1.417 Å and the one being broken is equal to 1.209 Å (see TS2 in Fig. 2a). Subsequently, the water molecule acting as an H bridge abstracts an H^+^ of the CH_4_ molecule and then returns a proton (H^+^) to the conjugated O site of the zeolite. The corresponding transition state for the C–C bond formation step displays the incoming C–C and the O–H bond formation with the distance of 1.820 and 1.805 Å, respectively (see Fig. 2b). It's noteworthy that the Gibbs energy barriers present values equal to 26.7–30.2 kcal mol^–1^, significantly lower than that for the MTO HCP reaction (*ca.* 40 kcal mol^–1^) in the steady state reaction,^36^,^40^ suggesting that the initial C–C bond formation is possibly on the synergistical BAS/LAS sites in ZSM-5 zeolite.

**Fig. 3:**
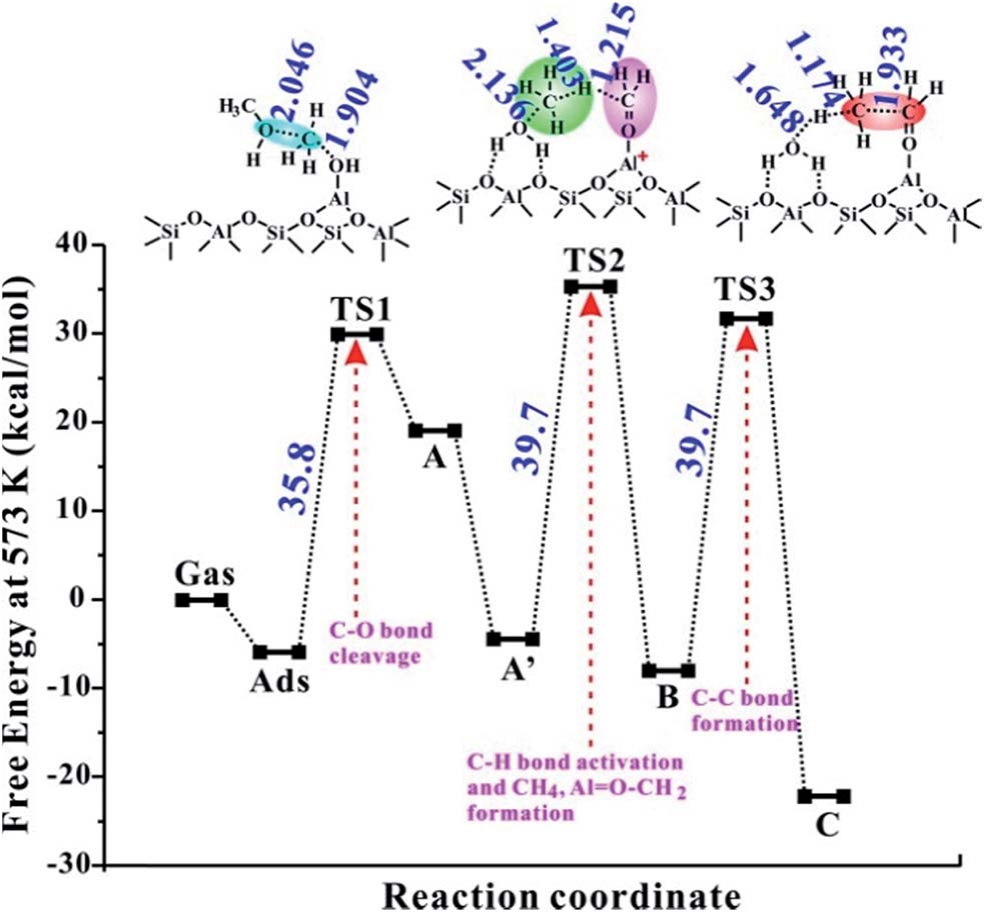
The reaction Gibbs free energy profile of the direct formation of the C–C bond following the newly proposed mechanism (see Scheme 1) at the Al(OH)_2_/BAS site over ZSM-5 zeolite at 573 K. The detailed reaction routes and definition of the abbreviations are shown Scheme 1. The main geometric parameters of the TS are given in Å.

Furthermore, the catalytic activities of other EFAl species inside ZSM-5 zeolite are also investigated theoretically and the corresponding activation barriers are listed in Table 1 and the energy profiles are provided in Fig. 3 and S7–S9.† It's noteworthy that the isolated AlOH^2+^ structure formation is at the expense of consuming proximal Brønsted acid protons. As shown in Table 1 and Fig. S7,† the C–O bond activation route with the transfer of the methoxy group on the isolated EFAl AlOH^2+^ site is associated with a barrier of 68.4 kcal mol^–1^, indicating that the reaction cannot occur during the MTO reaction. It is illustrated that the synergistical BAS/LAS sites were an indispensable factor to accelerate the DME reaction. The corresponding barriers of the C–C bond formation (Δ*G*_act1_, Δ*G*_act2_, and Δ*G*_act3_) are 35.8, 39.7 and 39.7 kcal mol^–1^, respectively (see Table 1 and Fig. 3), on the Al(OH)_2_/BAS site. It's noteworthy that the barrier of the rate-determination step (39.7 kcal mol^–1^) is close to that of in the MTO cycles (40 kcal mol^–1^).^36^ Therefore, the initial C–C bond is possibly formed by the synergism of Al(OH)_2_/Brønsted sites. However, compared with the barriers of AlOH (26.7–30.2 kcal mol^–1^), the relatively higher barriers apparently indicated the formation of kinetically less favorable Al(OH)_2_ species. While for the Al(OH)_3_/Brønsted sites, formation of Al–OCH_2_^+^ intermediates is unlikely because its barrier is as high as 56.7 kcal mol^–1^ in step 2 (see Table 1 and Fig. S8†). This trend is in good agreement with the experimental results for the Al_2_O_3_ samples that the Al(OH)_3_ species over fully hydrated Al_2_O_3_ surfaces are inactive for the C–H bond activation.^21^ For the isolated Al(OH)_2_^+^ (originated from terminal oxygen of AlOOH protonated by proximal Brønsted acid sites) at the ZSM-5 framework, the initial C–O bond activation is also kinetically prohibited with the barrier as high as 82.6 kcal mol^–1^ (see Table 1 and Fig. S9†). On the basis of the energy data for all the possible EFAl species inside ZSM-5 zeolite, it can be concluded that both AlOH and Al(OH)_2_ are effective active sites for the C–C bond formation, while, the C–C bond cannot be realized on the neutral species Al(OH)_3_ and isolated AlOH^2+^ and Al(OH)_2_^+^ species in the MTO reaction.

**Table 1:** Computed Gibbs free energy barriers (kcal mol^–1^) of the conventional and newly proposed methane–formaldehyde routes on the Brønsted acid sites and synergistic Brønsted/Lewis acid sites for the C–C bond formation in the MTO reaction at 573 K. The detailed reaction routes are shown in Schemes 1, S1 and S2 (see the ESI)

			Newly proposed mechanism					Conventional Brønsted		
			Isolated Lewis acid		Synergistic Brønsted/Lewis acid			Acid mechanism		
			AlOH^2+^	Al(OH)_2_^+^	AlOH^2+^/BAS	Al(OH)_2_^+^/BAS	Al(OH)_3_/BAS	TMO	DME	Methanol
ZSM-5	C–C formation	Δ*G*_act1_	68.4	82.6	26.7	35.8	40.6	26.8/13.6	35.7	33.3
		Δ*G*_act2_	—	—	29.8	39.7	56.7	23.5	23.5	23.5
		Δ*G*_act3_	—	—	30.2	39.7	—	41.8	41.8	41.8
	Ethene formation	Δ*G*_act4_			19.7					
		Δ*G*_act5_			32.4					
		Δ*G*_act6_			11.5					
SSZ-13	C–C formation	Δ*G*_act1_	82.8	58.5	54.4	30.6	25.0	30.5/19.0	37	28.2
		Δ*G*_act2_	—	—	—	40.2	33.0	45.2	45.2	45.2
		Δ*G*_act3_	—	—	—	17.8	26.2	45.4	45.4	45.4
	Ethene formation	Δ*G*_act4_					20.2			
		Δ*G*_act5_					16.8			
		Δ*G*_act6_					23.7			

### The effective route for ethene formation

**Fig. 4:**
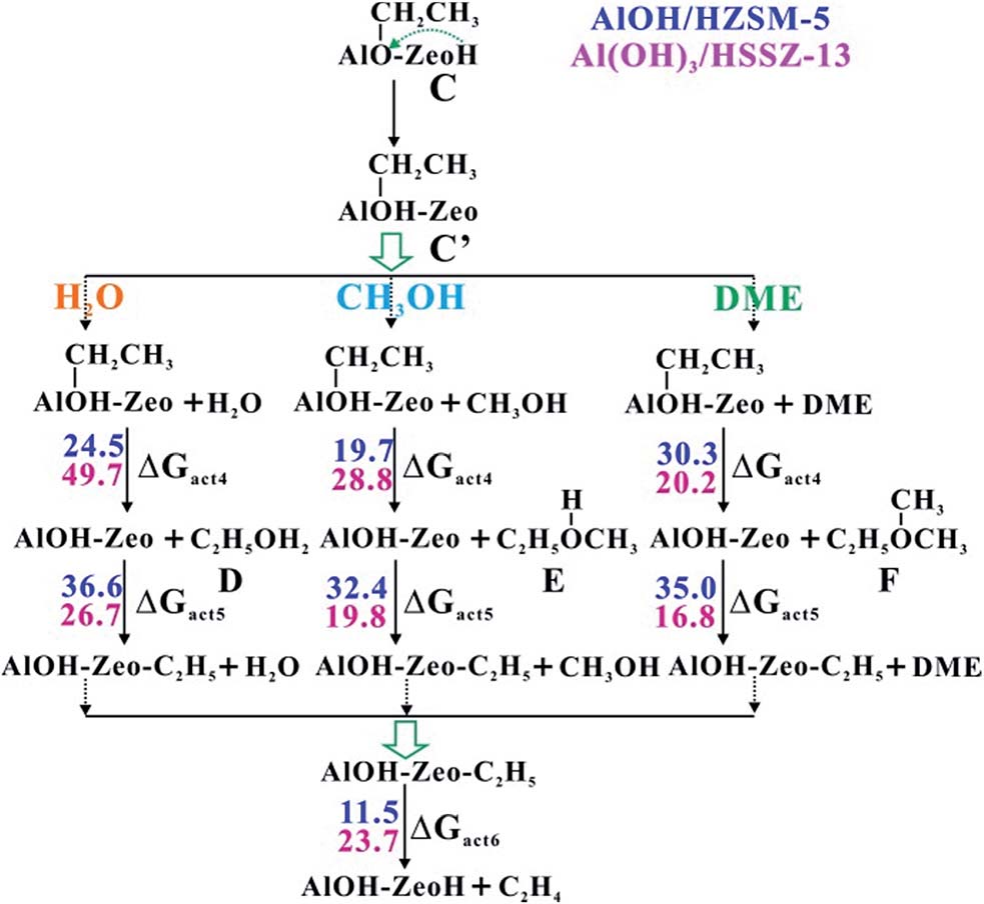
The proposed route for ethene formation from intermediate C over AlOH/HZSM-5 and Al(OH)_3_/HSSZ-13 (see Scheme S2†). The Gibbs free energy barriers (Δ*G*_act_, in kcal mol^–1^) for each elementary step at 573 K have been listed (D, represents the protonated ethanol; E, represents the protonated CH_3_CH_2_OCH_3_; F, represents CH_3_CH_2_O^+^(CH_3_)_2_ oxonium).

It is well known that ethene plays a crucial role in the MTO reaction, which can act not only as the olefin product but also as the key intermediate for HCP formation. Thus, the next important case is the pathway of ethene formation from the Al-bound ethoxide (C). Three possible routes involving H_2_O, methanol and DME are considered in this work. The reactions contain three elementary steps: (1) abstracting the ethyl group from the Al-bound ethoxide intermediate and regeneration of LAS; (2) yielding the surface ethoxide; (3) formation of ethene and regeneration of BAS (see Scheme S2†). As shown in Fig. 4, the barriers of the rate-determining steps in the three routes over ZSM-5 zeolite are 36.6 (H_2_O), 32.4 (methanol) and 35.0 kcal mol^–1^ (DME), respectively. The low activation barriers reveal that all three routes are feasible. Noticeably, the DME route is related to the CH_3_CH_2_O^+^(CH_3_)_2_ oxonium ion (F, in Fig. 4). This is in agreement with the previous studies by Liu *et al.* that the oxonium ion could be captured during the initial period of the MTO reaction by the *in situ* SSNMR experiment and it acted as a paramount intermediate during the initial ethene formation.^22^ Among the three routes, the CH_3_OH-mediated route prevails, which could be ascribed to the well fit dimension of protonated CH_3_CH_2_OCH_3_ (E) with the ZSM-5 pore structure. Overall, it is apparently indicated that the DME reaction over the AlOH/BAS site of ZSM-5 could produce alkenes readily, and then, the alkenes can generate HCP species to initiate the MTO cycles self-sustained in the steady state.

### The initial C–C bond formation over SSZ-13 zeolite

SSZ-13 zeolite with the CHA topology structure is another extensively used zeolite catalyst for the MTO reaction due to the pore selectivity, which possesses a cage-like pore structure with an effective pore diameter of 7.31 Å.^41^ Thus, the initial alkene formations involving the Al–OCH_2_^+^ intermediate over SSZ-13 zeolite are also investigated in this work. In contrast to ZSM-5 zeolite, the AlOH/BAS in SSZ-13 results in a larger barrier for the initial C–O bond activation of DME (54.4 kcal mol^–1^, see Fig. S10†). Such a large barrier could be ascribed to the severe deformation of the AlOH group from the adsorbate to the transition state (Fig. S10†). The energy barriers at 573 K (Table 1, Fig. 1 and S10–S14 in the ESI†) show that the Al(OH)_3_/BAS site over SSZ-13 zeolite is more effective for catalyzing the C–C bond formation among the five species. The adsorption energy of DME at the Al(OH)_3_/BAS site over SSZ-13 zeolite is –5.9 kcal mol^–1^ similar to that over SAPO-34 (–8.1 kcal mol^–1^).^19^ The energy barriers of the C–C bond formation are shown in Fig. 1, and the complete structure changes during the C–C bond formation are shown in Fig. S15 and Table S3†. The energy barriers are in the range of 25.0–33.0 kcal mol^–1^ similar to that over AlOH/HZSM-5 zeolite (Fig. 1 and Fig. S5†), indicating that the C–C bond could be easily formed over H-SSZ-13 zeolite in the induction stage. The calculated results show that the C–C bond formation between CH_4_ and Al–OCH_2_^+^*via* TS3 readily occurred over ZSM-5 and SSZ-13 zeolites. Different from ZSM-5, which has a high Si/Al ratio and prevents the proximity of two EFAL centers, the low Si/Al ratio in SSZ-13 results in the proximity of two EFAl centers. Therefore, the C–C bond formation between CH_4_ and Al–OCH_2_^+^*via* another neighboring Lewis acid site is also explored over SSZ-13 zeolite. The transition state displays the C–C formation and the C–H bond breakage with the distance of 1.970 and 1.227 Å (see Fig. S16b†), which is similar to the transition state *via* the bridge H_2_O (see Fig. 2d). The calculated energy barrier is 32.4 kcal mol^–1^ at 573 K, demonstrating the possibility of C–C bond formation through other neighboring Lewis acid sites in the low Si/Al ratio zeolite. Additionally, the ethene formation routes at the Al(OH)_3_/BAS site over SSZ-13 zeolite are also investigated. Compared with the three routes for ethene formation (Fig. 4), the DME route is preferable, different from ZSM-5 zeolite (methanol route). The calculated barrier for ethoxide formation through the CH_3_CH_2_O^+^(CH_3_)_2_ oxonium (F) intermediate is 16.8 kcal mol^–1^ lower than that in ZSM-5 (35.0 kcal mol^–1^), which could be ascribed to the larger zeolite pore dimension of SSZ-13 (*D*_i_ = 7.31 Å) than ZSM-5 (*D*_i_ = 6.30 Å).^41^ Obviously, the ethene formation is favored kinetically, with the barrier of 23.7 kcal mol^–1^ for the rate-determination step, signifying that the reaction readily occurs at 573 K.

### The driving force for the CH_4_ activation

**Fig. 5:**
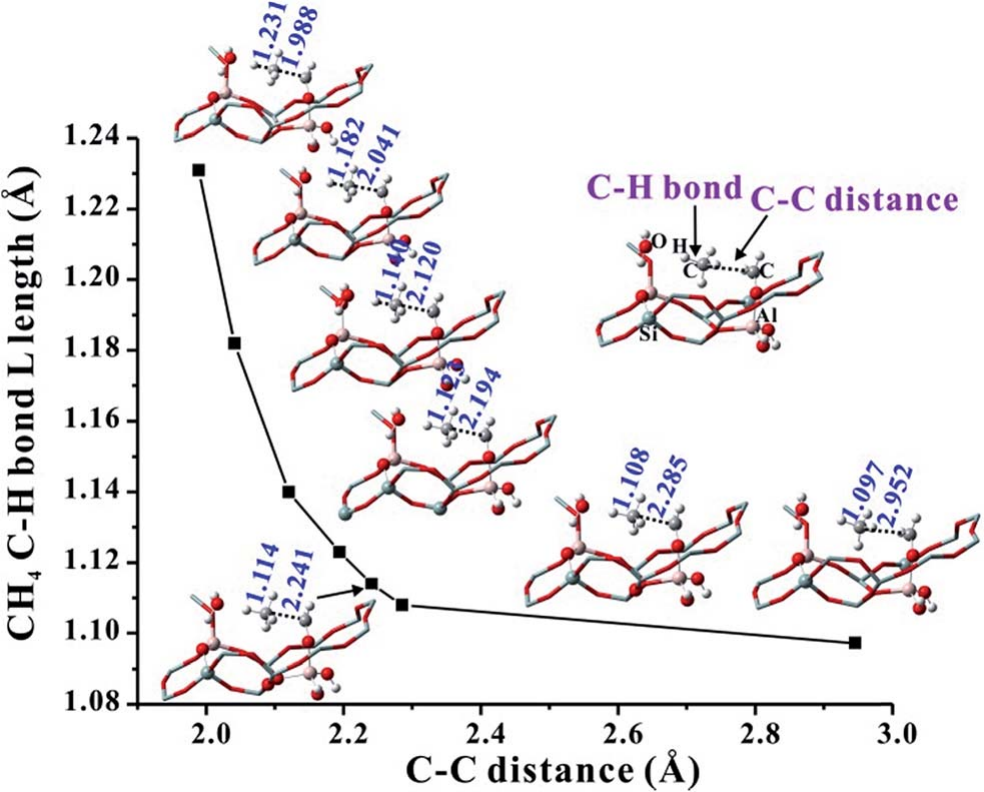
Variation of the C–H bond length of CH_4_ corresponding to the change of the CH_4_ and Al–OCH_2_^+^ distance (C–C distance) over Al(OH)_3_/HSSZ-13.

**Fig. 6:**
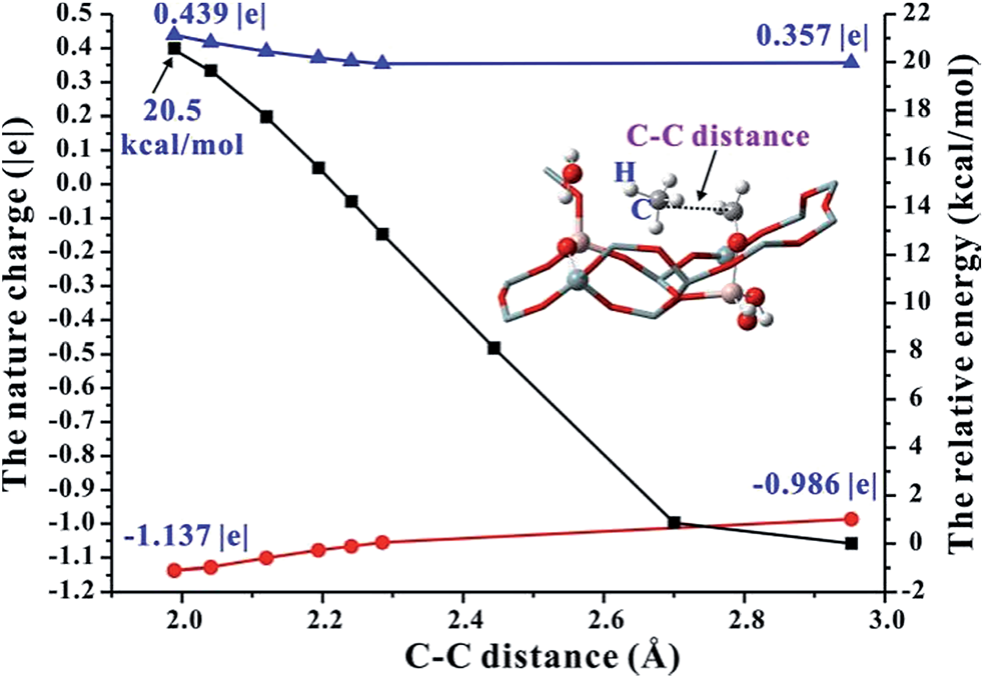
The nature bond charge (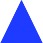 for C atom in CH_4_; 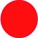 for H atom in CH_4_) and energy variation (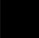) corresponding to the change of the CH_4_ and Al–OCH_2_^+^ distance (C–C distance) over Al(OH)_3_/HSSZ-13.

On the basis of the aforementioned facts, the coupling of CH_4_ and Al–OCH_2_^+^ intermediate is crucial for the C–C bond formation in the MTO reaction. As is well known, the CH_4_ molecule is very inert and the C–H bond dissociation energy is as high as 105 kcal mol^–1^.^42^ Therefore, the CH_4_ activation mechanism on the synergistical Brønsted/Lewis acid sites should be investigated in detail. It is noteworthy that the CH_4_ molecule could be polarized by a strong nucleophile (*i.e.*, HCHO) to lead to the C–C bond coupling between CH_4_ and HCHO with a barrier at 44.0 kcal mol^–1^ through the traditional methane–formaldehyde route inside ZSM-5 zeolite.^16^,^27^ Additionally, such a methane–formaldehyde route for the C–C bond formation on the BAS is also investigated in this work. As shown in Fig. 1, the barrier of the initial C–C bond formation between CH_4_ and HCHO on the zeolite BAS site is 41.8–45.4 kcal mol^–1^, which is in agreement with the previous work.^16^ Compared with neutral HCHO (the positive charge of C atom, 0.221|*e*|), the HCHO bound to the Lewis acid site (*e.g.*, Al–OCH_2_^+^, positive charge of C atom, 0.357|*e*|) would be more electrophilic and susceptible to polarizing CH_4_, and consequently a relatively lower barrier for the C–C bond formation will be obtained. In order to explore the driving force of the C–C bond formation step, the C–H bond length of CH_4_ approaching the strong electrophilic Al–OCH_2_^+^ species has also been investigated. It is observed that the C–H bond of CH_4_ is gradually activated as illustrated in Fig. 5 that the C–H bond length of CH_4_ is elongated from 1.097 (adsorbed state) to 1.231 Å (transition state) with the decreasing intermolecular distance between CH_4_ and Al–OCH_2_^+^ (C–C distance). The potential energy curve in Fig. 6 also illustrates that such an approach of CH_4_ to Al–OCH_2_^+^ will overcome energy no more than 21 kcal mol^–1^, demonstrating that this process is feasible during the MTO reaction. Additionally, the negative charge of the C atom in CH_4_ gradually decreases (from –0.986 |*e*| to –1.137 |*e*|) and the H positive charge gradually increases (from 0.357 to 0.439 |*e*|) as the C–C distance decreases from 2.952 to 1.989 Å, indicating the gradually increasing nucleophilic attack on CH_4_ by Al–OCH_2_^+^ and the deprotonation of CH_4_ to the bridge H_2_O (see Fig. 6). Thus, it can be concluded that the strong electrophilic character of the Al–COH_2_^+^ intermediate is the driving force for the CH_4_ activation and C–C bond formation in our work.

### Propene formation following the newly proposed mechanism

**Fig. 7:**
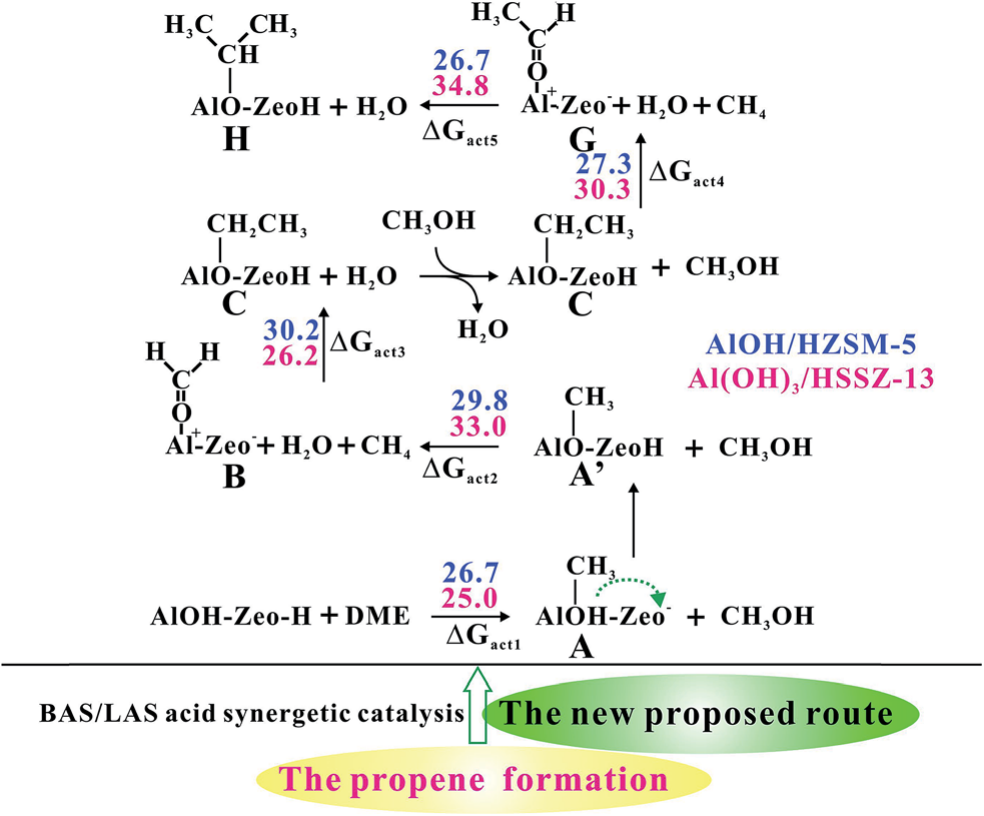
The newly proposed methane–formaldehyde routes for Al-bound propoxide (Al–O–CH(CH_3_)_2_) formation at synergistical BAS/LAS sites in the MTO reaction over the AlOH/HZSM-5 site and Al(OH)_3_/HSSZ-13 catalyst. The Gibbs free energy barriers (Δ*G*_act_, in kcal mol^–1^) for each step have been listed at 573 K. The detailed reaction routes are shown in Scheme S4† (A, represents the Al–OH-bound methyl (Al–OHCH_3_); A′, represents the Al-bound methoxide (Al–OCH_3_); B, represents Al–OCH_2_^+^ intermediate; C, represents Al-bound ethoxide (Al–O–CH_2_CH_3_); G, represents Al–OCHCH_3_^+^ intermediate (CH_3_CHO bound the Al^3+^ centre); H, represents Al-bound propoxide (Al–O–CH(CH_3_)_2_)).

The experimental work by Kondo demonstrated that the propene also serve as the initial product of MTO reactions by infrared (IR) spectroscopy.^43^ In terms of the newly proposed route, propene can be generated independent of the ethene route (Scheme S3†), which coincides with Kondo's experimental work.^43^ As illustrated in Fig. 7 and S17,† the Al-bound ethoxide (important intermediate for ethene formation) can further react with methanol and give rise to the intermediate G (Al–COHCH_3_^+^) formation with a barrier of 27.3 kcal mol^–1^ over ZSM-5 zeolite. The intermediate G can be considered as acetaldehyde (CH_3_CHO) adsorbed on the Al^3+^ Lewis acid site. The strong electrophilic character of the Al^3+^ Lewis acid site is conducive to the second C–C bond formation between the Al–OCHCH_3_^+^ and CH_4_, and results in the intermediate H (Al–O–CH(CH_3_)_2_) formation with the barrier of 26.7 kcal mol^–1^. It's noteworthy that intermediate H is an important species for propene formation. Subsequently, H can readily produce propene through the CH_3_OH-mediated route with the highest barrier of 35.7 kcal mol^–1^ (see Fig. S18†). Thus, it can be concluded that, similar to ethene formation, the DME reaction over the AlOH/BAS site of ZSM-5 could produce propene as well. The direct propene formation following the newly proposed mechanism over Al(OH)_3_/HSSZ-13 zeolite is also investigated (see Fig. 7 and S18†). The calculated barrier is 11.6–34.8 kcal mol^–1^, indicating that the formation of propene is also feasible over Al(OH)_3_/HSSZ-13. Overall, in addition to ethene, propene also serves as the initial product during the MTO in H-ZSM-5 and HSSZ-13 zeolites.

### Experimental evidence of the newly proposed mechanism

In this work, we theoretically identified a new methane–formaldehyde pathway for the initial alkene formation induced by synergistic interaction of BAS/LAS inside zeolite frameworks. It is noteworthy that extensive experimental work existed in previous work to support this new route. On the one hand, the synergistical Lewis/Brønsted acid activated center (*e.g.*, Al(OH)_3_ and AlOH EFAl species in close proximity to BAS) has been determined by the advanced NMR approach in the ZSM-5 and other zeolite catalysts.^23^,^24^ On the other hand, the CH_4_, HCHO, Al–OCH_3_ and oxonium ion intermediates involving the new mechanism also have been observed in the MTO catalytic process. For example, it's experimentally observed that CH_4_ could be produced during the initial period of the MTO reaction by Hutchings and coworkers.^15^ Lercher *et al.* indicated that the HCHO could be generated in ZSM-5 zeolite, and it's further found that the LAS could accelerate the formation of HCHO.^39^ On the basis of the ^13^C NMR experiment, the Al–OCH_3_ intermediate has also been detected on the surface AlOH site by Philippe *et al.*^21^ Furthermore, it's illustrated that the reaction activity following this new proposed mechanism is considerably enhanced compared to the conventional pathways at the isolated Brønsted or Lewis acid sites. As shown in Table 1, the barriers for the initial ethene formation have significantly decreased from 41.8–45.4 kcal mol^–1^ at the isolated BAS to 32.4–33.0 kcal mol^–1^ at the synergistical BAS/LAS sites over ZSM-5 and SSZ-13 zeolites. Moreover, the new mechanism is also more effective at the synergistical BAS/LAS sites than at the isolated LAS sites of γ-Al_2_O_3_ surface (with the barrier of 38 kcal mol^–1^).^21^ Consequently, the synergistic effect of the adjacent BAS/LAS sites in the zeolite catalysts could significantly decrease the energy barriers of the initial ethene formation in the MTO reaction through the new methane–formaldehyde route. On the other hand, it's demonstrated that the effective EFAl structures (AlOH^2+^ or Al(OH)_3_) and detailed reaction pathways strongly determined by the zeolite unique framework properties.

### Concept of synergy of Brønsted and Lewis acid sites on other catalytic reactions

The DFT calculations in our work give direct theoretical evidence that the synergy of EFAl and Brønsted acid sites in zeolite catalysts could alter the reaction mechanism, and thus strongly reduce the activation barrier of the initial C–C bond formation in the MTO reaction. Therefore, it provided a paradigm for the synergy of Lewis acid sites (EFAl) and Brønsted acid sites in zeolite catalysts and facilitated the catalytic reactions with the complete mechanism calculations. It's noteworthy that such a synergistic concept has been directly or indirectly suggested in the catalytic experiments as well. For instance, Schallmoser *et al.* showed that the strong BAS vicinity of EFAl displayed a rate enhancement in alkane cracking.^44^ Lercher *et al.* demonstrated that the synergy of EFAl and BAS could promote the production of the aromatic and light alkanes during the MTH (methanol to hydrocarbon) reaction.^39^ Huang *et al.* also proved that the cooperativity of BAS and EFAl significantly improved the yield of acrolein from the selective glycerol dehydration.^45^ Besides the EFAl, interaction of other extra-framework metal cations such as La^3+^ and Ga^2+^ with BAS could improve the catalytic activity as well. Lercher *et al.* showed that the cooperative effect of La^3+^ cations and the presence of BAS sites promoted catalytic isomerization, cracking, and alkylation of alkanes.^46^ Hensen *et al.* indicated that the synergy between Ga and BAS had the higher activity with relatively weak coke formation in the *n*-heptane cracking reaction.^47^ Despite the promoting effect of BAS/LAS synergy being widely explored, little mechanistic investigation for such synergistic effect on the pathways has been done. In principle, three possible ways of BAS/LAS synergy promote the zeolite catalytic performances. (I) A typical feature of the BAS/LAS synergistic effect is enhancing the strength of BAS resulting in the higher catalytic reactivity, as illustrated by the recent DFT theoretical calculations and catalytic experiments for alkane activations.^44^,^48^. (II) LAS can also act as a active center for hydrocarbon activation and transformation, and thus the presence of the Lewis acid site in the zeolite surface will provide an opportunity for the Lewis acid-catalyzed pathways distinct from Brønsted acid catalysis.^39^,^46^ (III) The synergy of proximal Lewis and Brønsted acid sites play a full role in the catalytic process resulting in the enhancement of the catalytic activity. Our calculation work brings new atomic-scale insights into understanding the detailed catalytic mechanism involved in BAS and LAS sites by the DFT calculation. The quantitative understanding of the reaction mechanism is key to design better and more stable BAS/LAS catalysts, and gives a clear blue print for material synthesis of new highly effective catalysts.

## Conclusions

In this contribution, the initial C–C bond formation during the initial stage of MTO process *via* a new methane–formaldehyde pathway on zeolite LAS/BAS was identified theoretically. For the first time, a formaldehyde-analogue (Al–OCH_2_^+^) intermediate, originated from a hydride abstraction from a surface Al–OCH_3_ species has been recognized to be the crucial intermediate for the initial C–C bond formation in MTO process over zeolite. The calculated Gibbs free energy barrier shows that the strong electrophilic character of the formaldehyde-analogue intermediate can strongly accelerate the C–C bond formation with CH_4_ and the overall reaction process is energy favorable. The proposed pathway in this contribution shows for the first time the initial ethene formation involved in various intermediate species observed in the previous experimental work, such as Al–OCH_3_, CH_4_, HCHO and oxonium ions. Additionally, this contribution also proves the different mechanism of the initial C–C bond formation with systematic calculation of all the active sites in ZSM-5 and SSZ-13, which is very important for demonstrating the structure–performance correlation for the MTO reaction.

Furthermore, this contribution gives direct evidence that the synergy of LAS and BAS in zeolite catalysts could facilitate catalytic reactions with complete mechanism calculations, and provide a good paradigm to determine the active sites and mechanisms of heterogeneous catalysis using high level DFT calculations.

## Conflicts of interest

The authors declare no competing financial interests.

## Supplementary Material

Supplementary information
