# Supplementary material for: Brønsted/Lewis acid sites synergistically promote the initial C–C bond formation in the MTO reaction
Source: Chem Sci. 2018 Jun 27;9(31):6470–9. doi: 10.1039/c8sc02302f (PMC6115684; doi:10.1039/c8sc02302f)
Supplement: Supplementary file 1 [file SC-009-C8SC02302F-s001.pdf]

Electronic Supplementary Material (ESI) for Chemical Science.
This journal is © The Royal Society of Chemistry 2018

Supporting Information for

Brønsted/Lewis Acid Synergistically Promote the Initial C-C Bond Formation in MTO Reaction

Yueying Chu, Xianfeng Yi, Chengbin Li, Xianyong Sun, and Anmin Zheng*

State Key Laboratory of Magnetic Resonance and Atomic and Molecular Physics, National Center for Magnetic Resonance in Wuhan, Key Laboratory of Magnetic Resonance in Biological Systems, Wuhan Institute of Physics and Mathematics, Chinese Academy of Sciences, Wuhan 430071, P. R. China.

E-mail: zhenganm@wipm.ac.cn

List of Contents

Scheme S1. The conventional methane-formaldehyde mechanism for the C-C bond formation. (Ads, represents the adsorbed state; TS, represents transition state; Int, represents the intermediate)

Scheme S2. The possible routes for the ethene formation from intermediate C formed in scheme 1.

Scheme S3. The new proposed mechanism for Al-bound propoxide (Al-O-CH(CH_3)$_2$) formation at the synergistical BAS/LAS sites over zeolite catalysts.

Figure S1. The optimized structures of AlOOH/HZSM-5 (a), AlO/HSSZ-13 (b) and AlOOH/HSSZ-13 and the corresponding protonated structures.

Figure S2. Representation of ZSM-5 (a, b, c, d and e) and SSZ-13 (f, g, h, i and j) zeolites with synergistic $AlOH^{2+}$/BAS (a, f), $Al(OH)_2^+$/BAS (c, h), $Al(OH)_3$/BAS (d, i) and isolated $AlOH^{2+}$ (b, g), $Al(OH)_2^+$ (e, j), respectively.

Figure S3 The optimized transition states of the DME formation over AlOH/HZSM-5 (a) and $Al(OH)_3$/HSSZ-13 (b). The main geometric parameters are labeled (in Å).

Figure S4 The optimized structures of DME adsorbed on the Brønsted site of AlOH/HZSM-5 zeolite. The main geometric parameters are labeled (in Å).

Figure S5. The reaction Gibbs free energy profile of C-C bond direct formation following the new proposed mechanism (See scheme 1) at the AlOH/BAS site over AlOH/HZSM-5 zeolite at 573 K.

Figure S6. The optimized structures of the intermediates and transition states for C-C bond direct formation following the new proposed mechanism (See scheme 1) at the AlOH/Brønsted site over ZSM-5 zeolite, and the corresponding structure parameters are listed in Table S2.

Figure S7. The reaction Gibbs free energy profile of the first step for the C-C bond direct formation following the new proposed mechanism (See scheme 1) at the isolated AlOH site over ZSM-5 zeolite at 573 K, and the corresponding optimized transition state structure.

Figure S8. The reaction Gibbs free energy profile of the first two steps for the C-C bond direct formation following the new proposed mechanism (See scheme 1) at the

$Al(OH)_3$/Brønsted site over ZSM-5 zeolite at 573 K, and the corresponding optimized transition state structures.

Figure S9. The reaction Gibbs free energy profile of the first step for the C-C bond direct formation following the new proposed mechanism (See scheme 1) at isolated $Al(OH)_2$ site over ZSM-5 zeolite at 573 K, and the corresponding optimized transition state structures.

Figure S10. The reaction Gibbs free energy profile of the first step for the C-C bond direct formation following the new proposed mechanism (See scheme 1) at the AlOH/ Brønsted site over SSZ-13 zeolite at 573 K, and the corresponding optimized transition state structure.

Figure S11. The reaction Gibbs free energy profile of the first step for the C-C bond direct formation following the new proposed mechanism (See scheme 1) at the isolated AlOH site over SSZ-13 zeolite at 573 K, and the corresponding optimized transition state structure.

Figure S12. The reaction Gibbs free energy profile for the C-C bond direct formation following the new proposed mechanism (See scheme 1) at the $Al(OH)_2$/Brønsted site over SSZ-13 zeolite at 573 K, and the corresponding optimized transition state structures.

Figure S13. The reaction Gibbs free energy profile for the C-C bond direct formation following the new proposed mechanism (See scheme 1) at the isolated $Al(OH)_2$ site over SSZ-13 zeolite at 573 K, and the corresponding transition state structures.

Figure S14. The reaction Gibbs free energy profile of C-C bond direct formation following the new proposed mechanism (See scheme 1) at the $Al(OH)_3$/BAS site over SSZ-13 zeolite at 573 K. The detail reaction routes and definition of the abbreviations were shown Scheme 1.

Figure S15. The optimized structures of the intermediates and transition states for C-C bond direct formation following the new proposed mechanism (See scheme 1) at the $Al(OH)_3$/Brønsted site over SSZ-13 zeolite.

Figure S16. The optimized reactant (a) and transition state structure (b) of the C-C bond formation between CH_4 and Al-OCH_2^+ via another neighbouring Lewis acid site over $Al(OH)_3$/Brønsted site over SSZ-13.

Figure S17. The optimized structures of the TS for $AlOCHCH_3^+$ (a, c) and Al-bound propoxide ($AlO-CH(CH_3)_2$) (b, d) formation over the synergistical AlOH/HZSM-5 (a, b) and $Al(OH)_3$/HSSZ-13 (c, b) sites in the zeolite catalysts.

Figure S18. The proposed route for propene formation form intermediate H over AlOH/HZSM-5 and $Al(OH)_3$/HSSZ-13.

Table S1. Calculational adsorption enthalpy (ΔH_{ads}), entropy (ΔS_{ads}) and Gibbs free energy (ΔG_{ads}) of dimethyl ether (DME) adsorbed on the five Lewis/Brønsted acid sites (BAS, LAS) of EFAL/HZSM-5 zeolites at 573 K.

Table S2 The main structure parameters (bond lengths (r) are labelled in Å, angles (<) are labelled in ^{o}) of the intermediates and transition states for C-C bond direct formation following the new proposed mechanism (See scheme 1) at the AlOH/Brønsted site over HZSM-5 zeolite, and the corresponding optimized structures are provided in Figure S6.

Table S3 The main structure parameters (bond lengths (r) are labelled in Å, angles (<) are labelled in ^{o}) of the intermediates and transition states for C-C bond direct formation following the new proposed mechanism (See scheme 1) at the $Al(OH)_3$/Brønsted site over HSSZ-13 zeolite, and the corresponding optimized structures are provided in Figure S15.

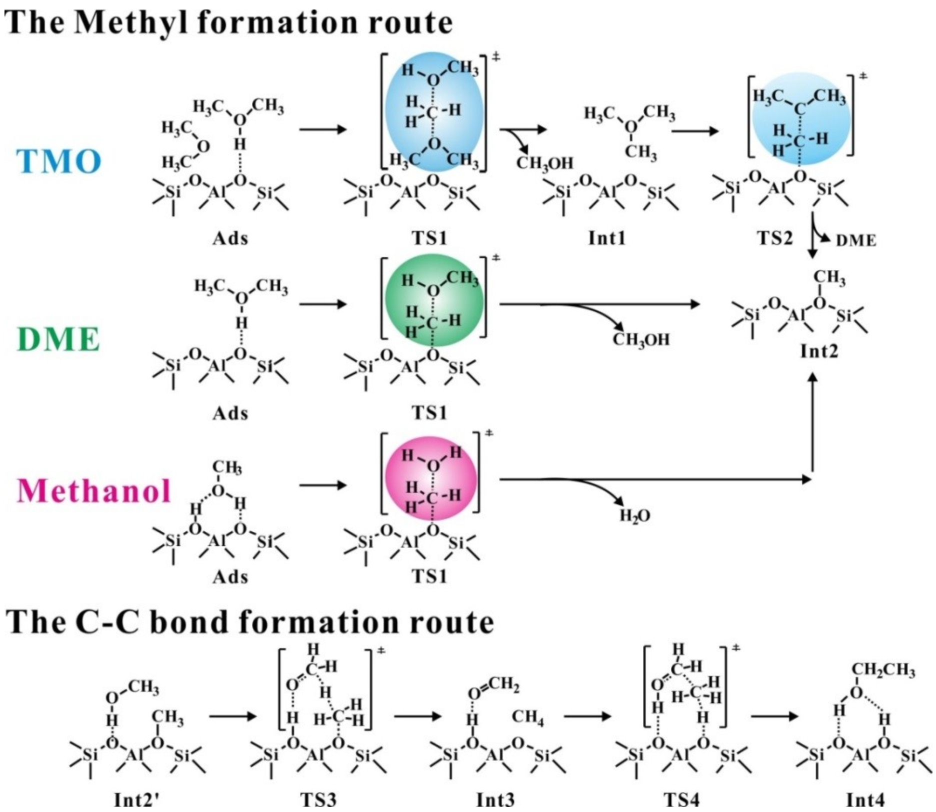

Scheme S1. The conventional methane-formaldehyde mechanism for the C-C bond formation. (Ads, represents the adsorbed state; TS, represents transition state; Int, represents the intermediate)

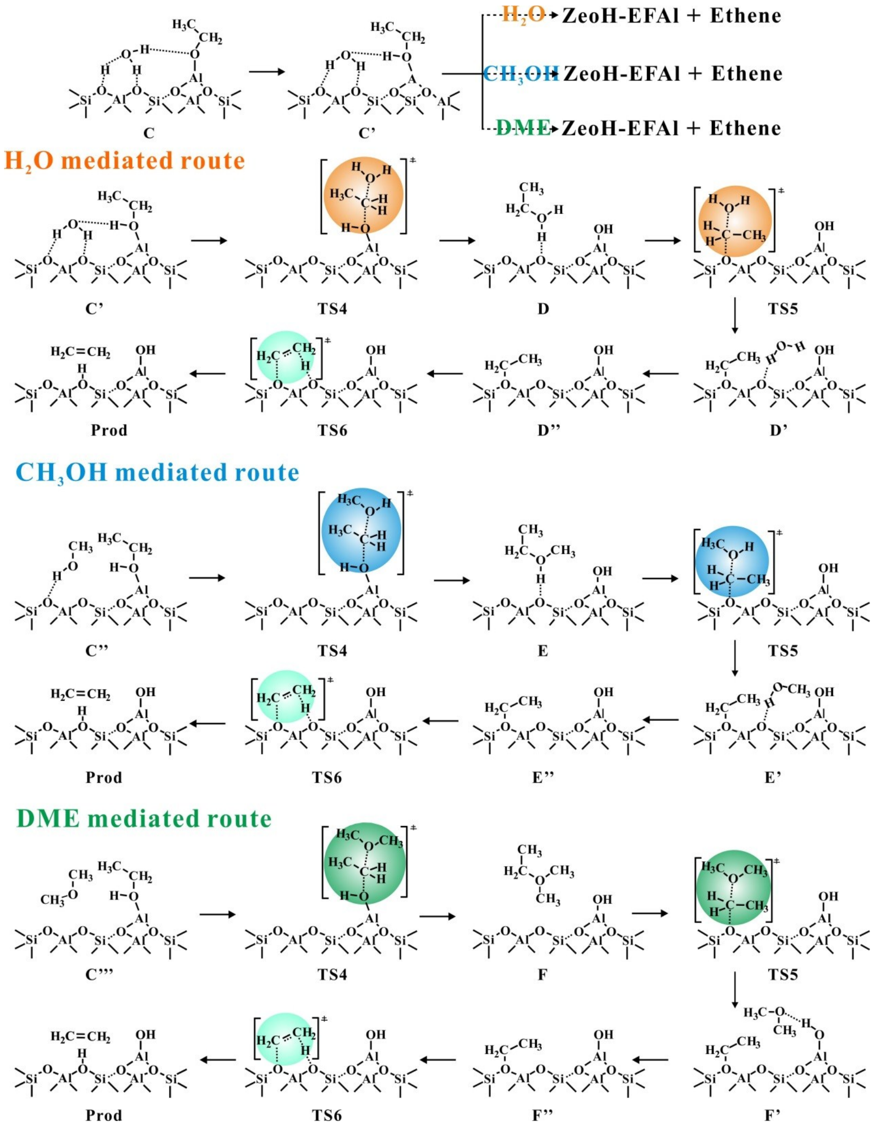

Scheme S2. The possible routes for the ethene formation from intermediate C formed in scheme 1. (C, represents Al-bound ethoxide (Al-O-CH_2CH_3); C’, represents the a H_2O adsorbed near the Al-$OHCH_2CH_3$; C’’, represents a methanol adsorbed near the Al-$OHCH_2CH_3$; C’’’, represents a DME adsorbed near the Al-$OHCH_2CH_3$; D, represents the protonated ethanol; D’, represents the surface ethoxide and adsorbed H_2O; D’’, represents the surface ethoxide in H_2O route; E, represents the protonated $CH_3CH_2OCH_3$; E’, represents the surface ethoxide and adsorbed methanol; E’’, represents the surface ethoxide in CH_3OH route; F, represents the $CH_3CH_2O^+(CH_3)_2$ oxonium; F’, represents the surface ethoxide and adsorbed DME; E’’, represents the surface ethoxide in DME route; TS, represents transition state; Prod, represents the ethene product)

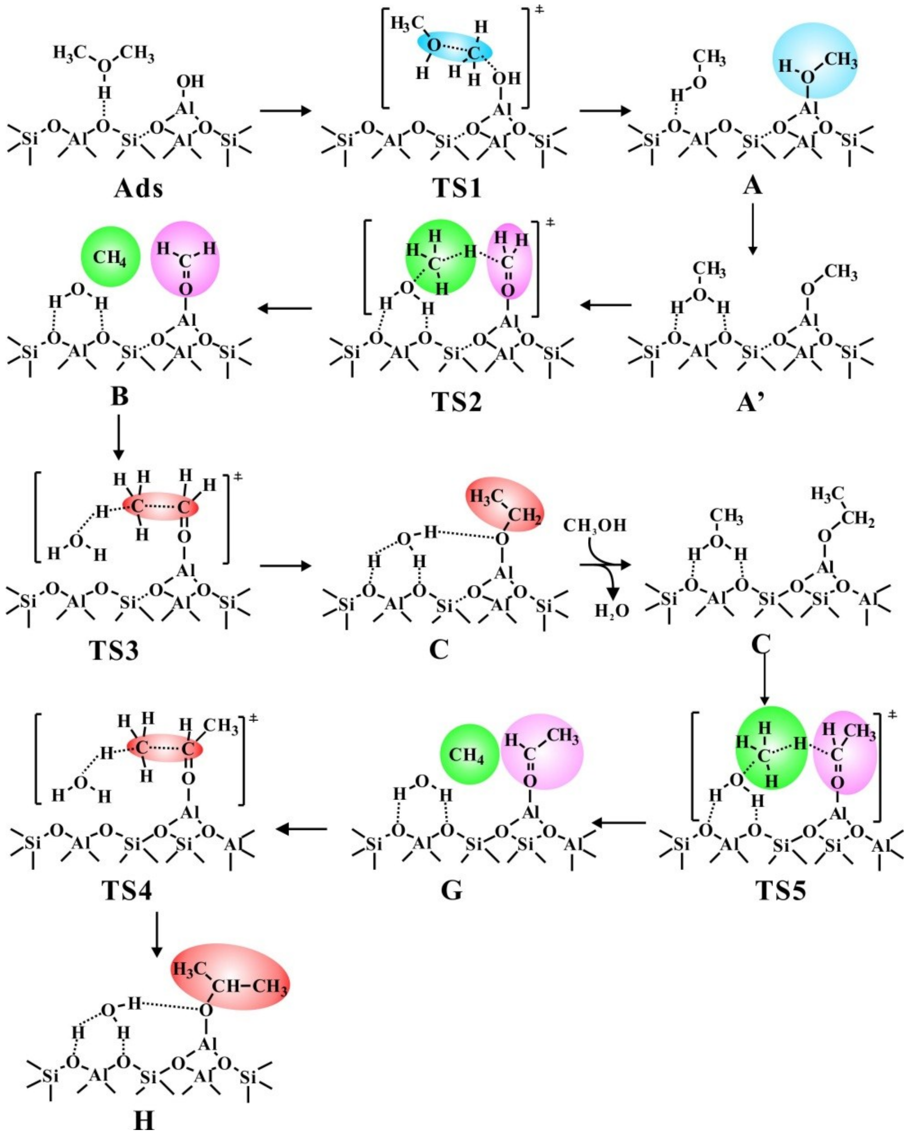

Scheme S3. The new proposed mechanism for Al-bound propoxide (Al-O-CH(CH_3)$_2$) formation at the synergistical BAS/LAS sites over zeolite catalysts. (Ads, represents the adsorbed DME; A, represents Al-OH-bound methyl (Al-OHCH_3); A', represents the Al-bound methoxide (Al-OCH_3); B, represents Al−OCH_2^+ intermediate; C, represents the Al-bound ethoxide (Al-O-CH_2CH_3); TS, represents transition state; G, represents Al−OCHCH_3^+ intermediate (CH_3CHO bound the Al^{3+} centre); H, represents Al-bound propoxide (Al-O-CH(CH_3)$_2$)

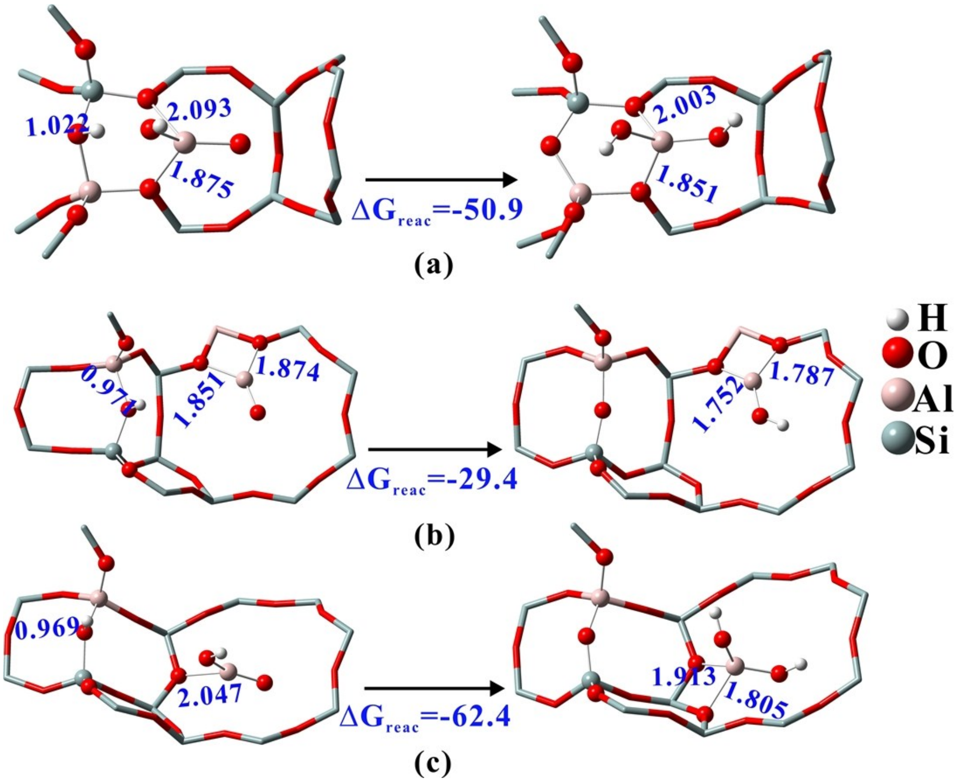

Figure S1. The optimized structures of AlOOH/HZSM-5 (a), AlO/HSSZ-13 (b) and AlOOH/HSSZ-13 and the corresponding protonated structures. It's observed that these EFAL structures with terminal oxygen Al=O groups were prone to form isolated $Al(OH)_2^+$ and $Al(OH)^{2+}$ EFAL structures through intramolecular proton transfer at the expenses of the nearby Brønsted acid site. The reactions Gibbs free energy at 573 K are given in kcal/mol. The main geometric parameters are labeled (in Å).

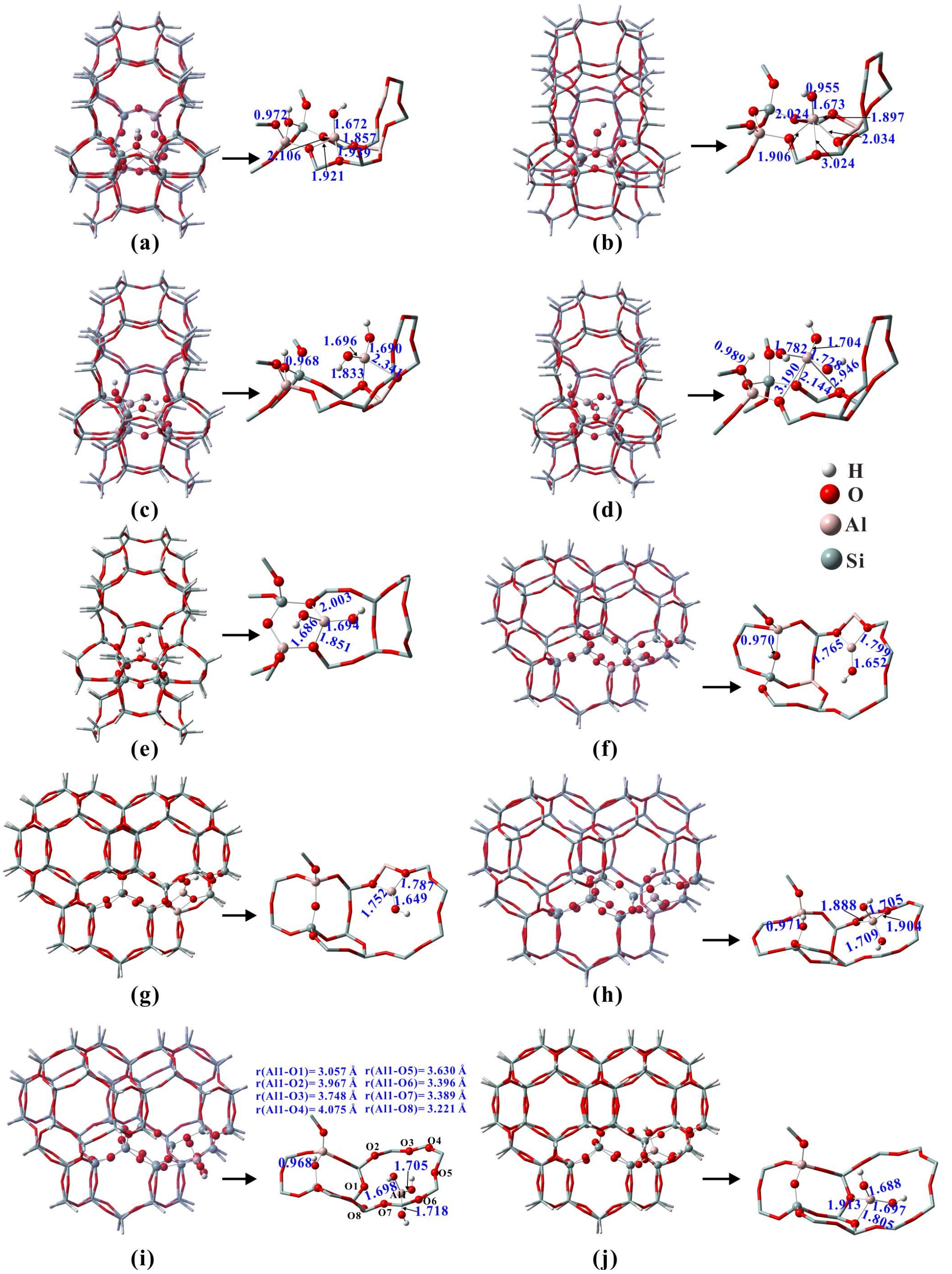

Figure S2. Representation of ZSM-5 (a, b, c, d and e) and SSZ-13 (f, g, h, i and j) zeolites with synergistic $AlOH^{2+}$/BAS (a, f), $Al(OH)_2^+$/BAS (c, h), $Al(OH)_3$/BAS (d, i) and isolated $AlOH^{2+}$ (b, g), $Al(OH)_2^+$ (e, j), respectively. The 10T for ZSM-5 and 14T for SSZ-13 cluster in the extended cluster models represented as ball and stick view was treated as high-layer atoms during the ONIOM calculations. The main geometric parameters are labeled (in Å).

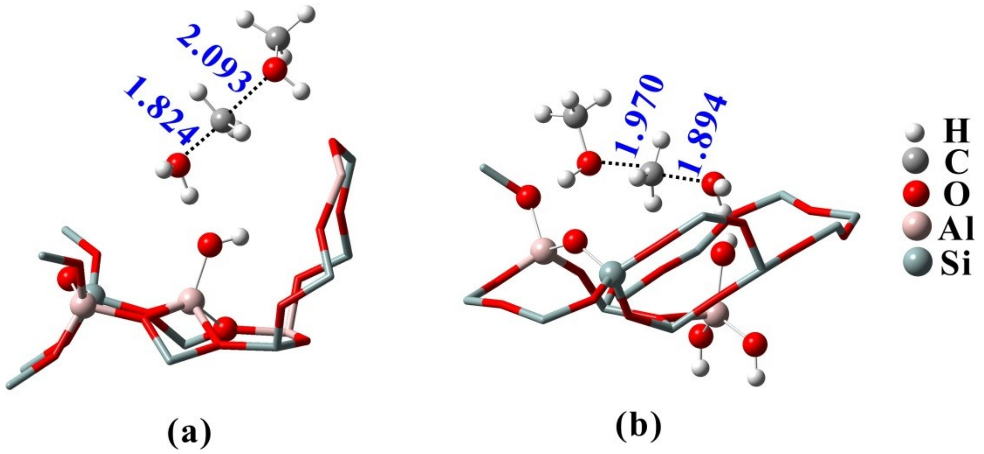

Figure S3 The optimized transition states of the DME formation over AlOH/HZSM-5 (a) and $Al(OH)_3$/HSSZ-13 (b). The main geometric parameters are labeled (in Å).

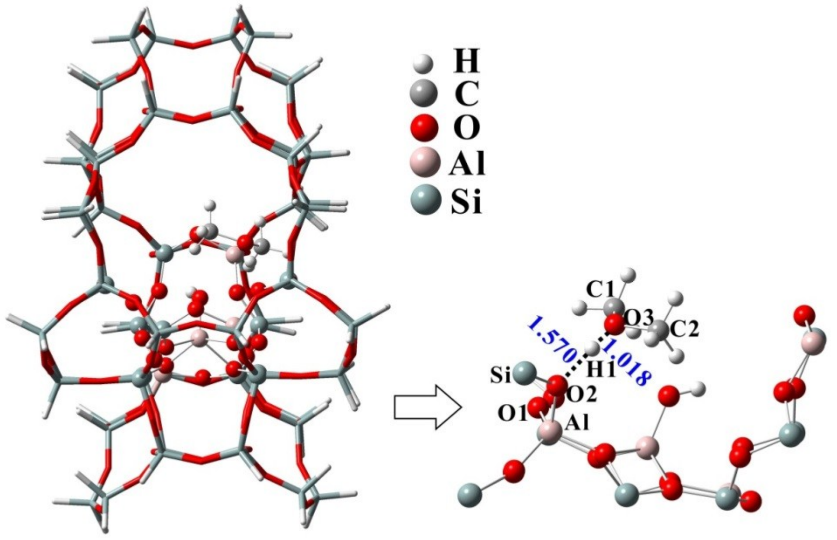

Figure S4 The optimized structures of DME adsorbed on the Brønsted site of AlOH/HZSM-5 zeolite. The main geometric parameters are labeled (in Å).

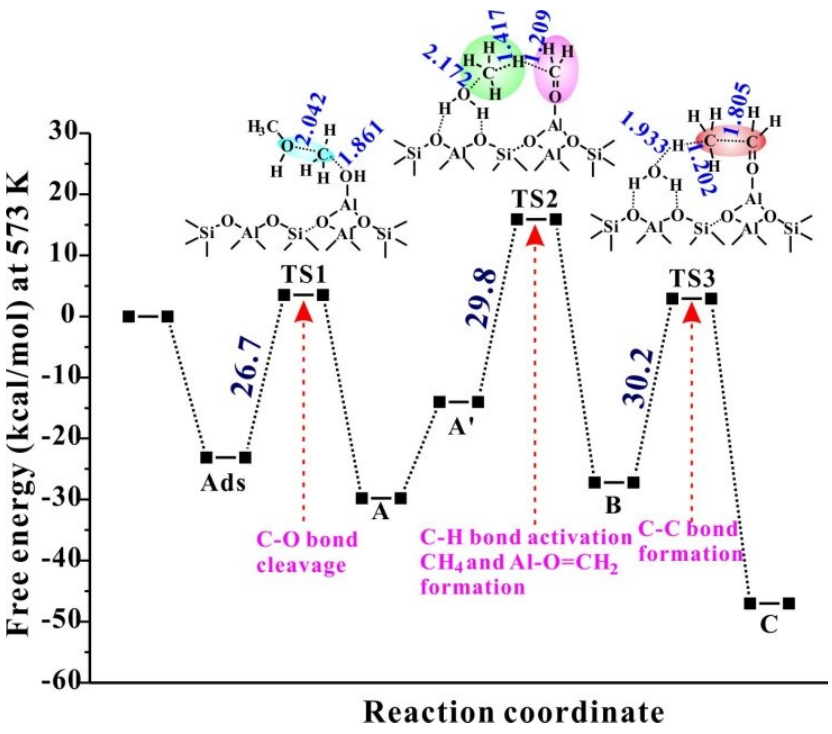

Figure S5. The reaction Gibbs free energy profile of C-C bond direct formation following the new proposed mechanism (See scheme 1) at the AlOH/BAS site over AlOH/HZSM-5 zeolite at 573 K. The detail reaction routes and definition of the abbreviations were shown Scheme 1. The main geometric parameters of the TS are given in Å.

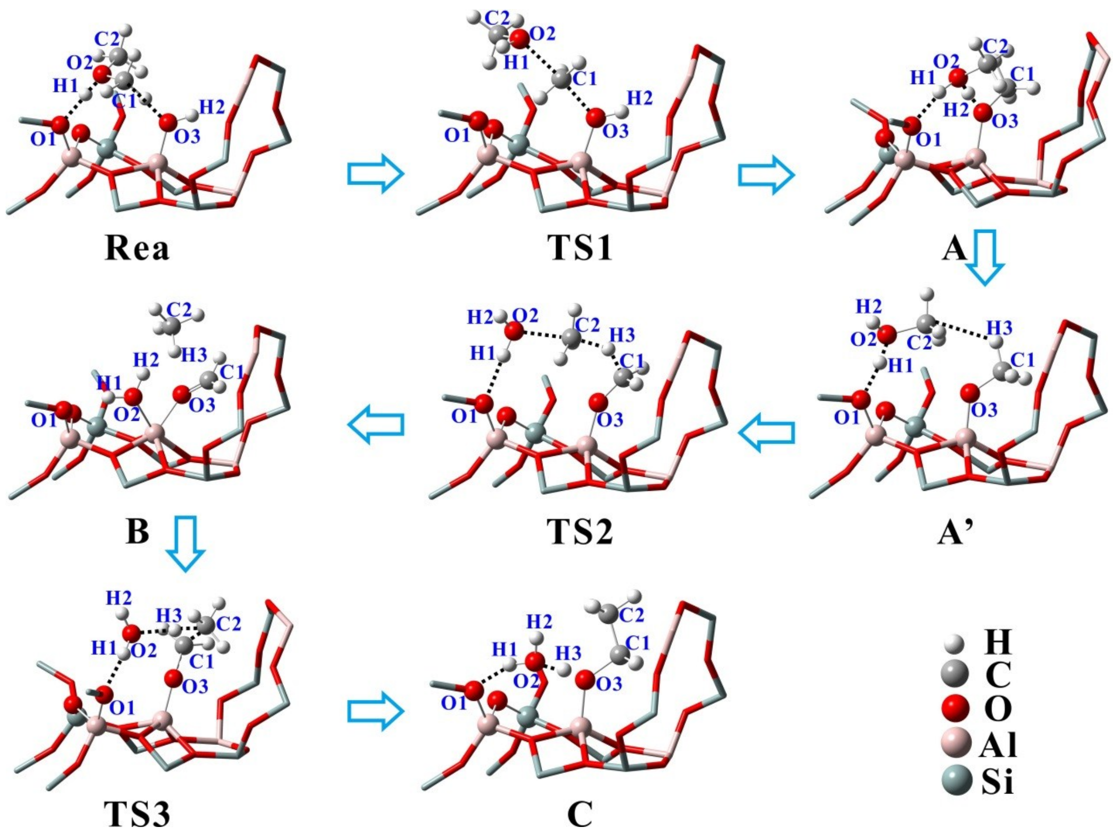

Figure S6. The optimized structures of the intermediates and transition states for C-C bond direct formation following the new proposed mechanism (See scheme 1) at the AlOH/Brønsted site over ZSM-5 zeolite, and the corresponding structure parameters are listed in Table S2.

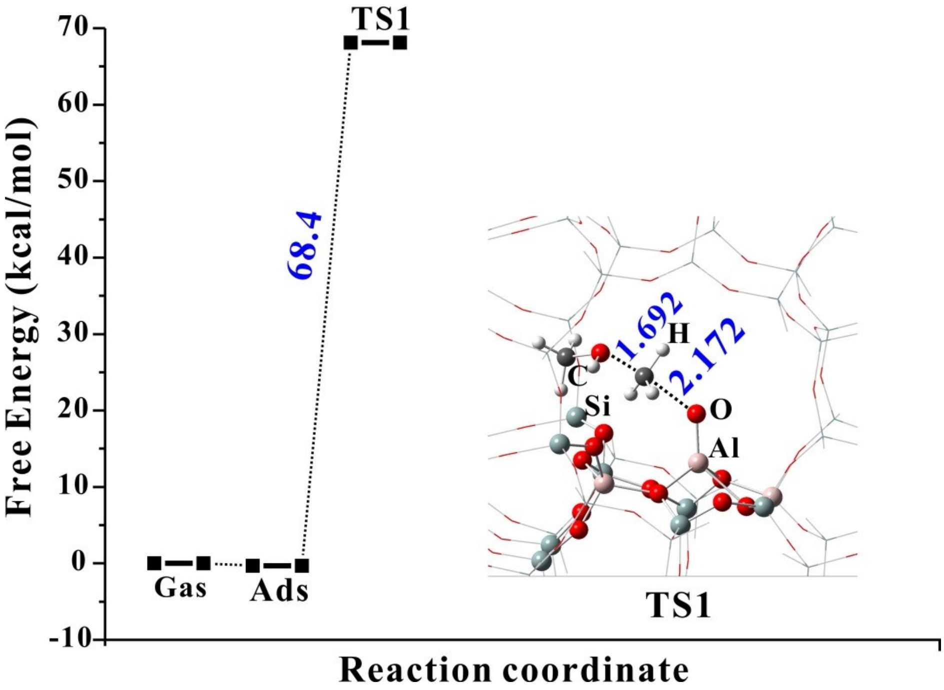

Figure S7. The reaction Gibbs free energy profile of the first step for the C-C bond direct formation following the new proposed mechanism (See scheme 1) at the isolated AlOH site over ZSM-5 zeolite at 573 K, and the corresponding optimized transition state structure. The main geometric parameters are labeled (in Å).

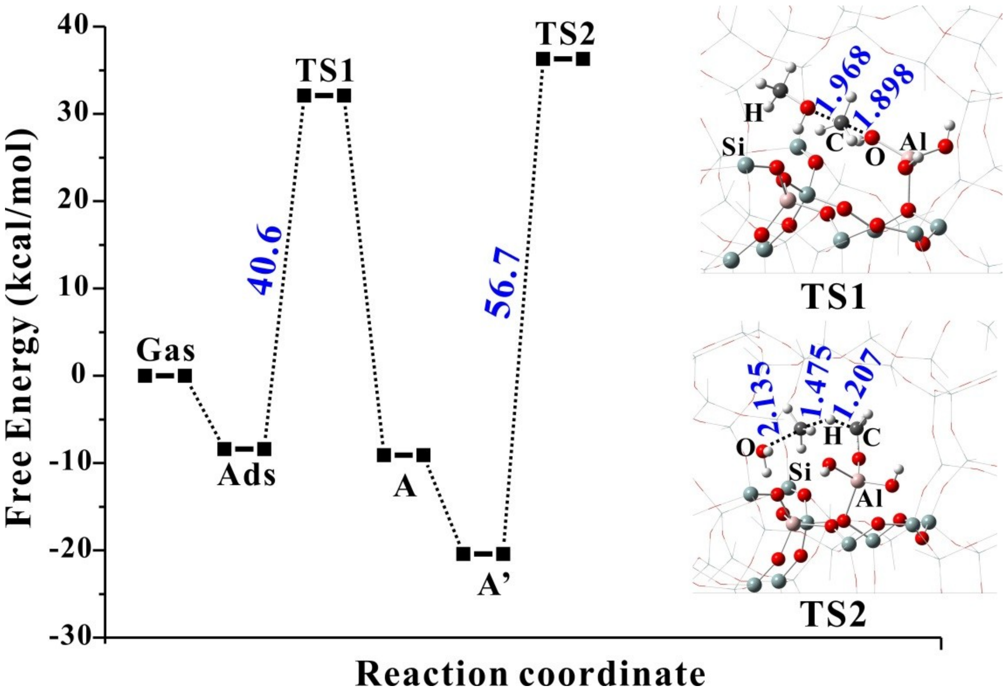

Figure S8. The reaction Gibbs free energy profile of the first two steps for the C-C bond direct formation following the new proposed mechanism (See scheme 1) at the $Al(OH)_3$/Brønsted site over ZSM-5 zeolite at 573 K, and the corresponding optimized transition state structures. The main geometric parameters are labeled (in Å).

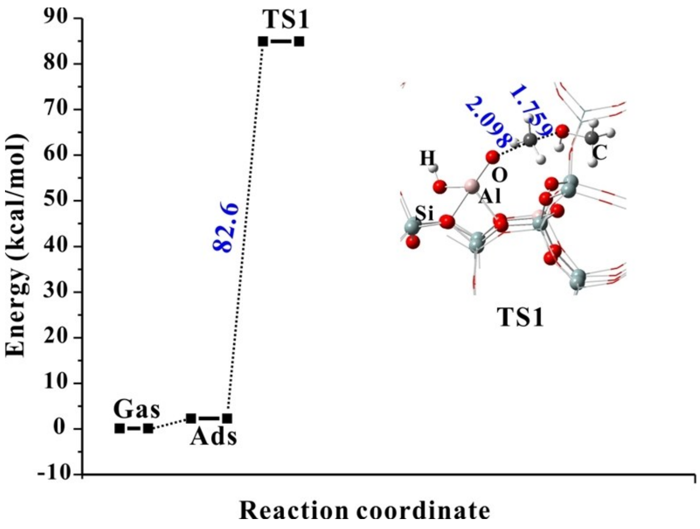

Figure S9. The reaction Gibbs free energy profile of the first step for the C-C bond direct formation following the new proposed mechanism (See scheme 1) at isolated $Al(OH)_2$ site over ZSM-5 zeolite at 573 K, and the corresponding optimized transition state structures. The main geometric parameters are labeled (in Å).

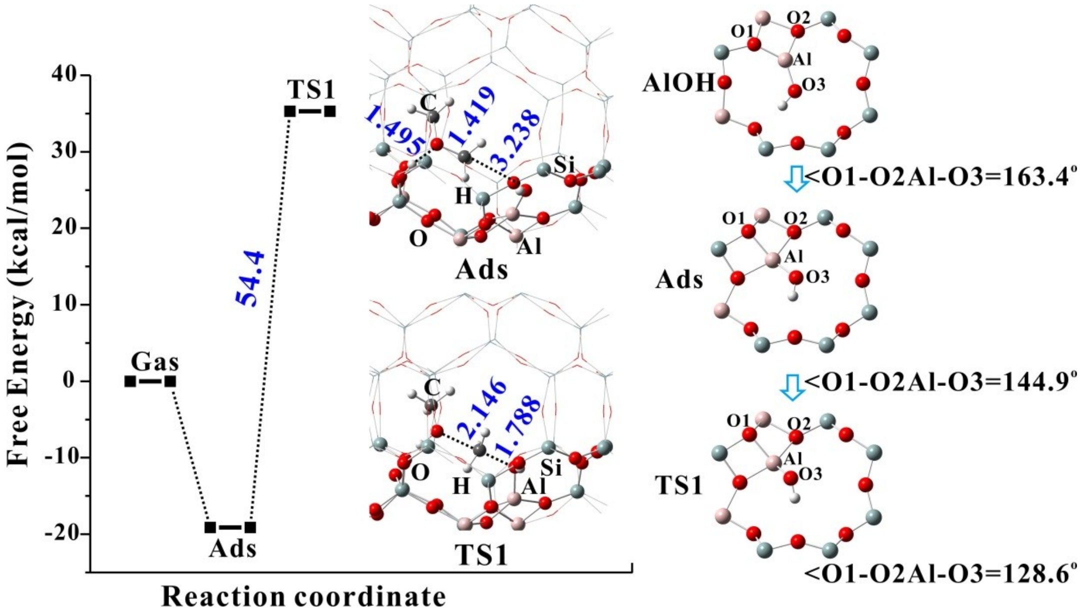

Figure S10. The reaction Gibbs free energy profile of the first step for the C-C bond direct formation following the new proposed mechanism (See scheme 1) at the AlOH/ Brønsted site over SSZ-13 zeolite at 573 K, and the corresponding optimized transition state structure. The main geometric parameters are labeled (in Å).

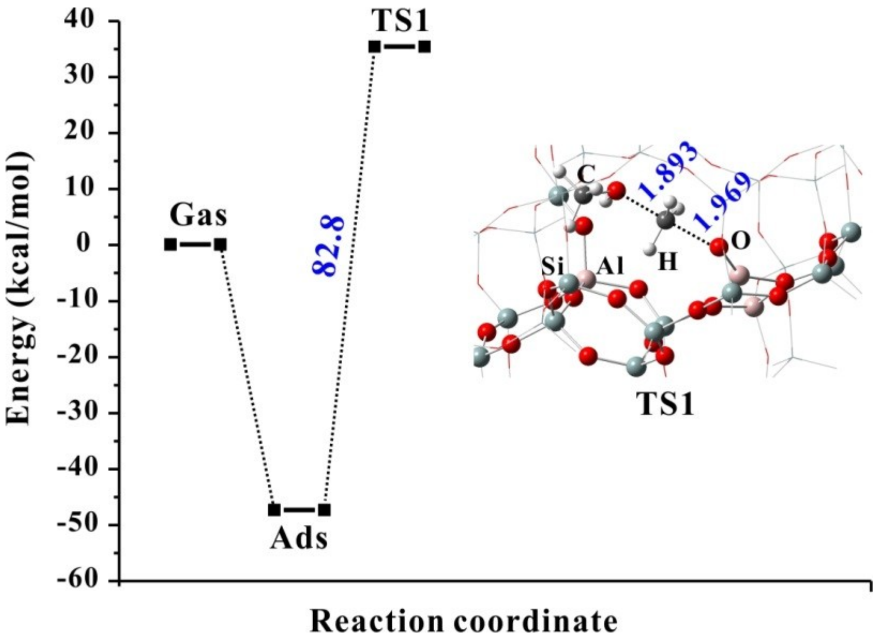

Figure S11. The reaction Gibbs free energy profile of the first step for the C-C bond direct formation following the new proposed mechanism (See scheme 1) at the isolated AlOH site over SSZ-13 zeolite at 573 K, and the corresponding optimized transition state structure. The main geometric parameters are labeled (in Å).

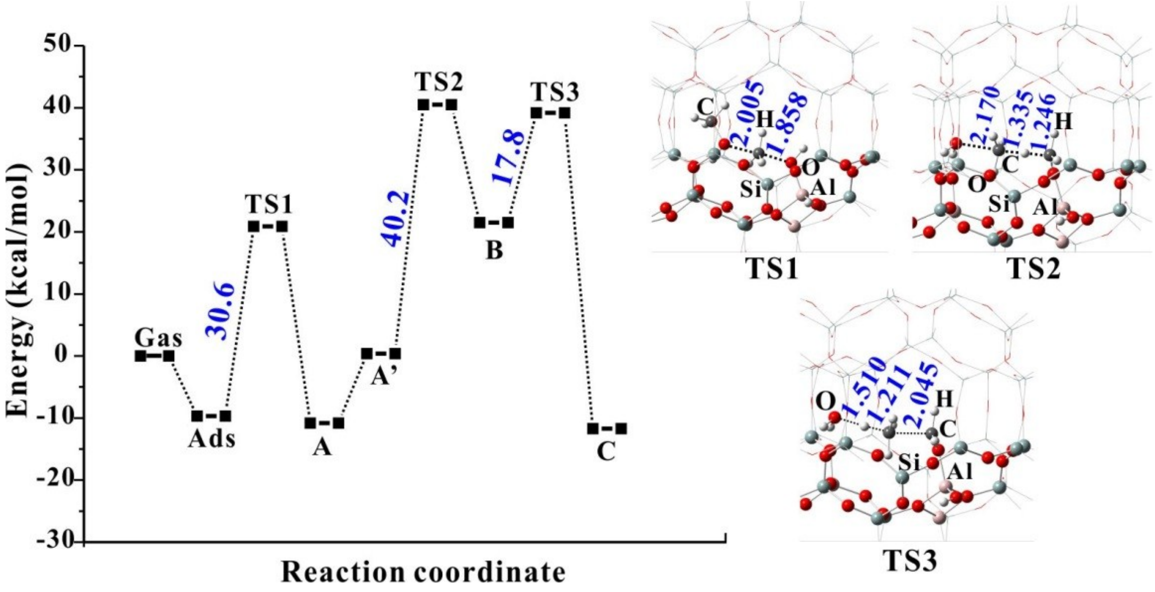

Figure S12. The reaction Gibbs free energy profile for the C-C bond direct formation following the new proposed mechanism (See scheme 1) at the $Al(OH)_2$/Brønsted site over SSZ-13 zeolite at 573 K, and the corresponding optimized transition state structures. The main geometric parameters are labeled (in Å).

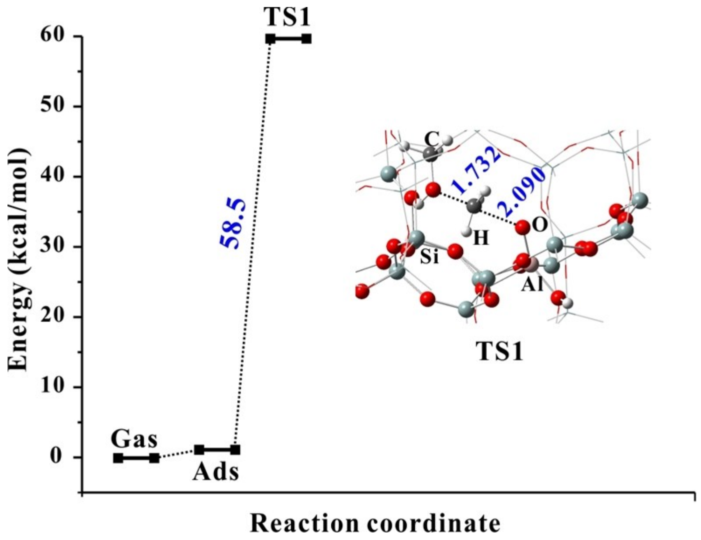

Figure S13. The reaction Gibbs free energy profile for the C-C bond direct formation following the new proposed mechanism (See scheme 1) at the isolated $Al(OH)_2$ site over SSZ-13 zeolite at 573 K, and the corresponding transition state structures. The main geometric parameters are labeled (in Å).

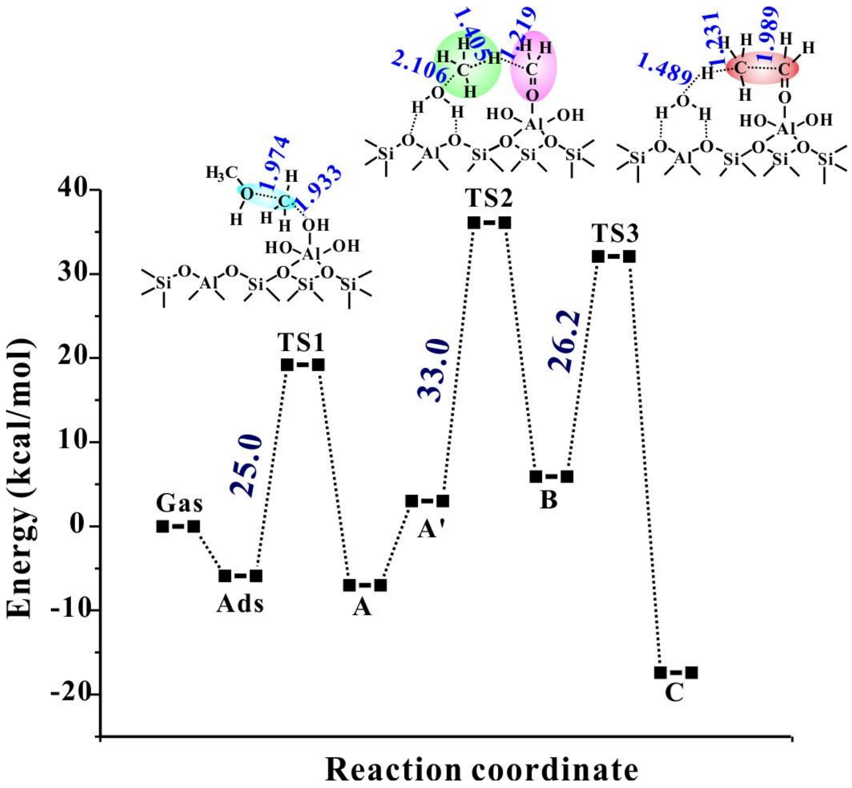

Figure S14. The reaction Gibbs free energy profile of C-C bond direct formation following the new proposed mechanism (See scheme 1) at the $Al(OH)_3$/BAS site over SSZ-13 zeolite at 573 K. The detail reaction routes and definition of the abbreviations were shown Scheme 1. The main geometric parameters of the TS are given in Å.

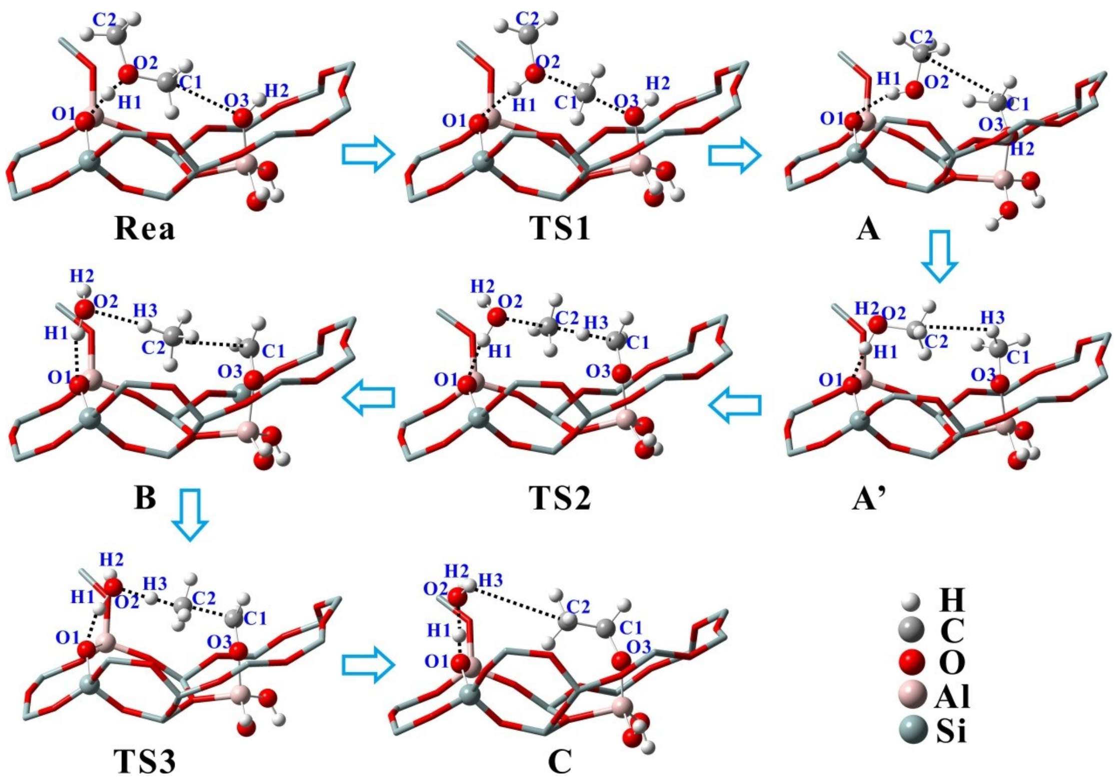

Figure S15. The optimized structures of the intermediates and transition states for C-C bond direct formation following the new proposed mechanism (See scheme 1) at the $Al(OH)_3$/Brønsted site over SSZ-13 zeolite, and the corresponding structure parameters are listed in Table S2.

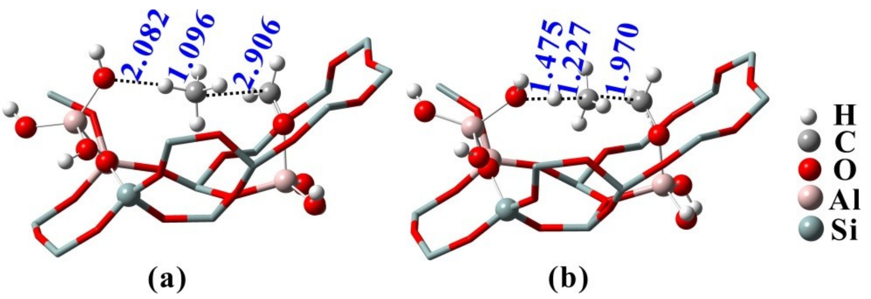

Figure S16 The optimized reactant (a) and transition state structure (b) of the C-C bond formation between CH_4 and Al-OCH_2^+ via another neighbouring Lewis acid site over $Al(OH)_3$/Brønsted site over SSZ-13. The main geometric parameters are labeled (in Å).

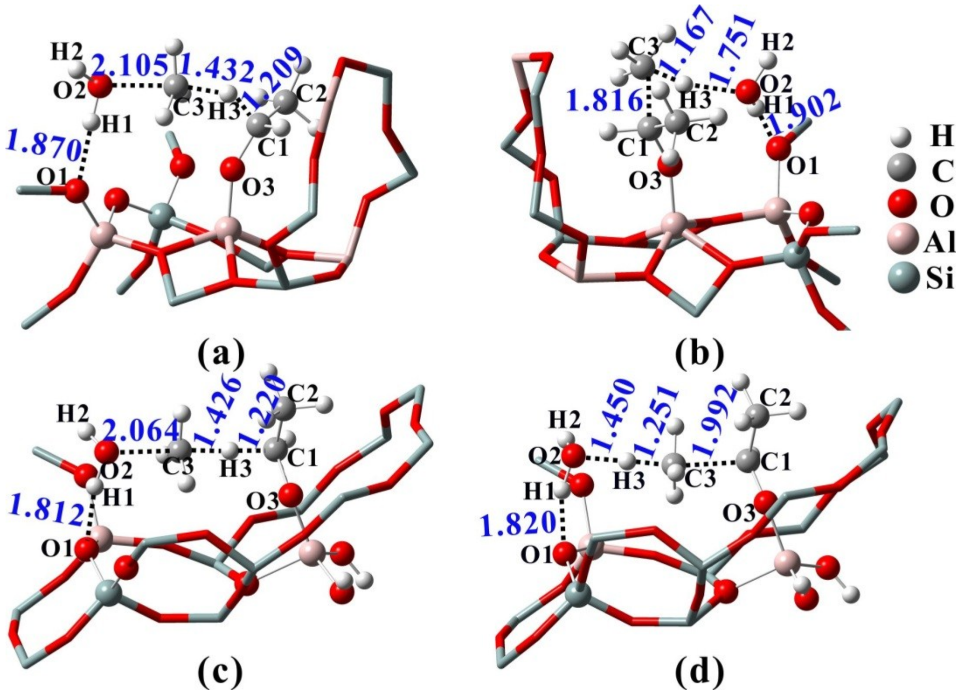

Figure S17. The optimized structures of the TS for $AlOCHCH_3^+$ (a, c) and Al-bound propoxide (AlO-$CH(CH_3)_2$) (b, d) formation over the synergistical AlOH/HZSM-5 (a, b) and $Al(OH)_3$/HSSZ-13 (c, b) sites in the zeolite catalysts. The main geometric parameters are given in Å.

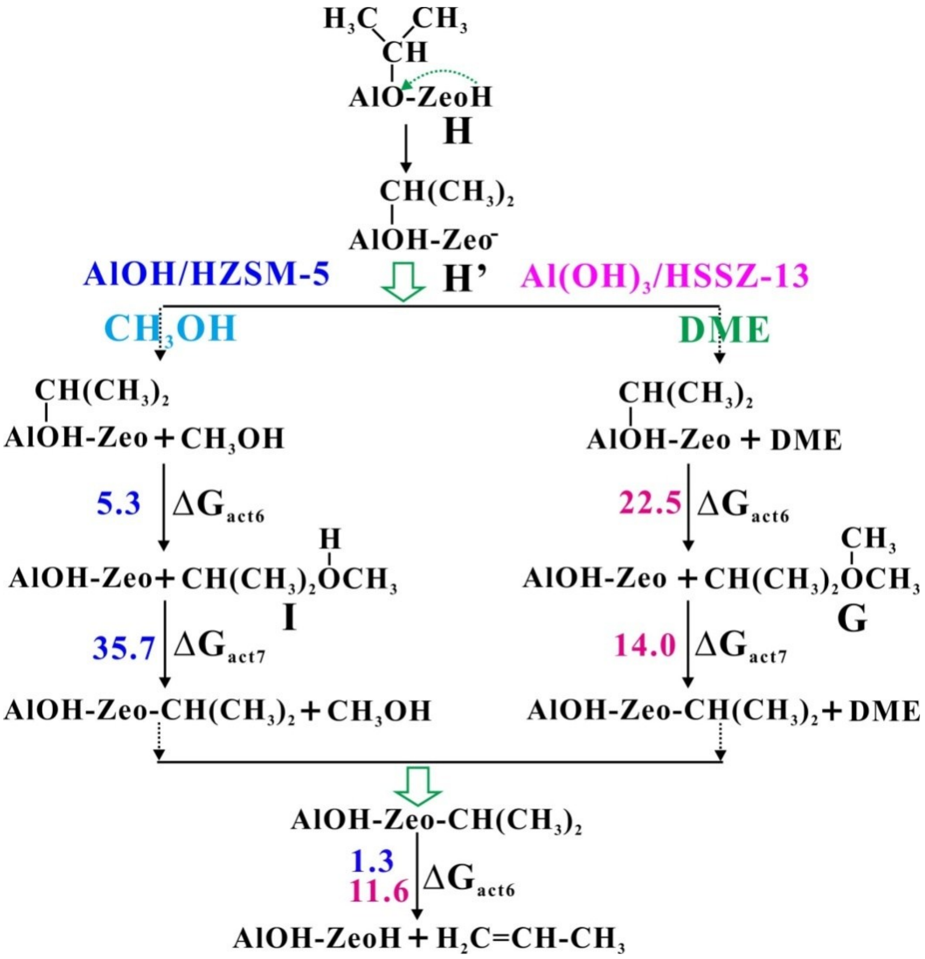

Figure S18. The proposed route for propene formation form intermediate H over AlOH/HZSM-5 and $Al(OH)_3$/HSSZ-13. The Gibbs free barriers (ΔG_{act}, in kcal/mol) for each elementary step have been listed at 573 K. (I, represents the protonated $CH_3CH_2OCH_3$; G, represents the $CH_3CH_2O^+(CH_3)_2$ oxonium)

Table S1. Calculational adsorption enthalpy (ΔH_{ads}), entropy (ΔS_{ads}) and Gibbs free energy (ΔG_{ads}) of dimethyl ether (DME) adsorbed on the five Lewis/Brønsted acid sites (BAS, LAS) of EFAL/HZSM-5 zeolites at 573 K.

	ΔH_{ads} (kcal/mol)	ΔS_{ads} (cal/mol.K)	ΔG_{ads} (kcal/mol)
Isolated $AlOH^{2+}$	-20.1	-34.6	-0.3
$Al(OH)^{2+}$	-49.2	-45.5	-23.2
$Al(OH)_2^+$	-29.8	-41.7	-5.9
$Al(OH)_3$	-32.0	-41.2	-8.4
Isolated $Al(OH)_2^+$	-19.6	-38.2	2.3

Table S2 The main structure parameters (bond lengths (r) are labelled in Å, angles (<) are labelled in º) of the intermediates and transition states for C-C bond direct formation following the new proposed mechanism (See scheme 1) at the AlOH/Brønsted site over HZSM-5 zeolite, and the corresponding optimized structures are provided in Figure S6.

	$r_{O1\text{-}H1}$	$r_{O2\text{-}H1}$	$r_{O2\text{-}C2}$	$r_{O2\text{-}C1}$	$r_{O3\text{-}C1}$	$r_{C1\text{-}H3}$	$r_{C2\text{-}H3}$	$r_{O2\text{-}H2}$	$r_{C1\text{-}C2}$	<O2C1O3	<O2C2H3	<C2H3C1	<O2H3C2	<C1H3C2
Rea	1.570	1.018	1.458	1.467	3.185					80.4				
TS1	2.805	0.970	1.429	2.042	1.861					173.9				
A	1.672	1.001	1.464	3.201	1.459					47.6				
A'	1.509	1.035	1.472		1.434	1.095	2.594	0.975			150.7	116.1		
TS2	1.920	0.976	2.172		1.354	1.209	1.417	0.966			169.5	128.5		
B	1.779	0.990	3.156		1.243		1.097	0.974	3.147				106.3	89.4
TS3	1.888	0.974			1.342		1.202	0.962	1.820				148.6	87.5
C	1.796	0.981			1.505		2.585	0.964	1.505				116.0	35.6

Table S3 The main structure parameters (bond lengths (r) are labelled in Å, angles (<) are labelled in ^{o}) of the intermediates and transition states for C-C bond direct formation following the new proposed mechanism (See scheme 1) at the $Al(OH)_3$/Brønsted site over HSSZ-13 zeolite, and the corresponding optimized structures are provided in Figure S15.

	r_{O1-H1}	r_{O2-H1}	r_{O2-C2}	r_{O2-C1}	r_{O3-C1}	r_{C1-H3}	r_{C2-H3}	r_{O2-H2}	r_{C1-C2}	<O2C1O3	<O2C2H3	<C2H3C1	<O2H3C2	<C1C2H3
Rea	1.384	1.077	1.452	1.456	2.988					164.8				
TS1	1.883	0.982	1.435	1.974	1.933					174.5				
A	1.982	0.970	1.421	3.334	1.457					108.1				
A'	1.672	1.006	1.459		1.397	1.100	2.887	1.048			164.8	106.4		
TS2	1.827	0.980	2.106		1.328	1.219	1.405	0.968			178.5	155.6		
B	1.938	0.966	3.263		1.229		1.097	0.963	2.952				167.5	166.2
TS3	1.891	0.977			1.290		1.231	0.972	1.989				177.2	176.4
C	1.059	1.398			1.400		3.910	0.969	1.527				73.1	168.7
